# Knowledge of the consequences of excessive alcohol consumption among emerging adult college students

**DOI:** 10.1590/1980-220X-REEUSP-2025-0088en

**Published:** 2025-08-22

**Authors:** Roselande Simon, Adriane Maria Netto de Oliveira, Cristiane Barros Marcos, Mara Regina Santos da Silva, Dominique Therrien, Patrícia Bitencourt Toscani Greco, Evy Nazon, Mara Regina Pombo Amaral, Émilen Vieira Simões

**Affiliations:** 1 Universidade Federal do Rio Grande, Programa de Pós-Graduação em Enfermagem, Rio Grande, RS, Brazil.; 2 Universidade Federal do Rio Grande, Instituto de Ciências Biológicas, Rio Grande, RS, Brazil.; 3 Université du Québec en Outaouais, Département des Sciences Infirmières, Gatineau, Québec, Canadá.

**Keywords:** Alcohol Drinking, Students, Human Health, Psychological Well-Being

## Abstract

**Objective:**

To examine the perception and knowledge of emerging adult college students regarding the consequences of excessive alcohol consumption on their biopsychosocial health and current and future well-being.

**Method:**

This exploratory, descriptive, qualitative study was conducted with 31 college students. A form with a QR code was made available online to provide information about the study. Data were collected through semi-structured interviews, transcribed using Turbo Scribe and the reports were analyzed using content analysis with the support of Atlas.ti software.

**Results:**

Participants demonstrated limited knowledge about what constitutes excessive alcohol consumption and its impact on health. These findings underscore the need for ongoing awareness and health education initiatives aimed at expanding knowledge and supporting informed decisions about alcohol use.

## INTRODUCTION

Alcohol is the most widely consumed psychoactive substance worldwide. Its use can range from moderate to excessive and harmful, potentially leading to alcohol dependence. According to the World Health Organization (WHO), any level of alcohol consumption poses risks to individuals’ health and well-being^(1)^. Studies indicate that alcohol use contributes to the global rise in non-communicable diseases, traffic accidents, domestic violence, physical and psychological violence, sexual abuse, and increased mortality. These outcomes result in higher healthcare expenditures as well as significant social and legal costs^(2,3)^. Consequently, alcohol consumption is recognized as a serious public health issue and a leading cause of preventable death.

Among all age groups, emerging adults exhibit the highest rates of alcohol consumption and the lowest rates of abstinence^(4,5)^. The concept of emerging adulthood was introduced by American psychologist Jeffrey Jensen Arnett in 2000 to describe the distinct developmental stage experienced by individuals between the ages of 18 and 25. Arnett identified five core characteristics of this phase. The first is identity exploration, during which young people seek to answer the question “Who am I?” in personal, relational, and professional domains. The second refers to instability, a characteristic explained by the fact that, during this stage, young people have not yet reached the stability typical of adult life. They are often exploring career paths, moving residences, and their goals and relationships tend to change several times until they can define more clearly what they want for themselves. The third is increased responsibility, as emerging adults begin laying the groundwork for their future quality of life, often leading to feelings of overload. The fourth characteristic is feeling in-between, capturing the ambivalence of no longer being adolescents but not yet fully adults. Finally, the fifth is a sense of possibilities, as this stage is marked by numerous opportunities to shape a fulfilling adult life^(6)^.

Among emerging adults, studies indicate that university students have a higher prevalence of alcohol consumption compared to their non-university peers. This may be attributed to their participation in activities that promote excessive drinking, such as parties, sporting events, and other social gatherings^(7,8)^. Gaining knowledge about the negative consequences of excessive alcohol use, as well as understanding what constitutes moderate consumption, may support the adoption of healthier behaviors. Additionally, recognizing risk factors associated with alcohol use can contribute to more informed and conscious decision-making^(9)^.

Information about the negative consequences of excessive alcohol consumption, as well as the patterns that define it, can help emerging adults evaluate whether such behavior is worth the associated risks. Access to this knowledge may facilitate the adoption of healthier daily habits aimed at preserving biopsychosocial health and well-being—not only for themselves but also for their families and communities^(10)^.

Studies show that peers significantly influence alcohol consumption among emerging adult college students^(7,8)^. When individuals interact with peers who drink in moderation, they are more likely to adopt and maintain similar behavior. Conversely, exposure to peers who engage in excessive drinking increases the likelihood of adopting the same pattern.

According to the latest WHO report, alcohol consumption accounted for approximately 2.6 million deaths worldwide in 2019. Additionally, 7% of the global population aged 15 and older has at least one alcohol-related mental disorder, with alcohol dependence being the most prevalent, affecting 3.7% of the population^(1)^. In Brazil, 50,000 hospitalizations related to alcohol use were recorded in 2023, and the alcohol-attributable mortality rate reached 33 per 100,000 inhabitants in 2022^(11)^.

In Brazil, the social costs of alcohol consumption were estimated at R$18.8 billion in 2019^(12)^. Given this evidence, promoting knowledge about the negative consequences of excessive consumption—and the thresholds that define it—constitutes a relevant and sustainable investment. With increased awareness, health, education, political, social, and public safety sectors can implement targeted campaigns to encourage moderate alcohol use. These efforts may contribute to the biopsychosocial health and overall well-being of the population, including emerging adult college students.

According to simulations by the Organization for Economic Cooperation and Development (OECD), the socioeconomic costs of alcohol consumption between 2020 and 2050 are projected to reach US$138 billion per year, including the loss of approximately 33 million workers and a 1.6% decline in GDP^(13)^. These figures underscore the relevance of this study. While alcohol consumption among university students has been widely researched, few studies have specifically examined emerging adults’ knowledge of what constitutes excessive consumption and its implications for health and well-being, both in the present and in the future. Therefore, this study aims to explore the perception and knowledge of emerging adult university students regarding the consequences of excessive alcohol consumption for biopsychosocial health and current and future well-being.

## METHODS

### Study Design

This study is part of the master’s thesis “*Transição para Adultos Emergentes e o Consumo de Bebidas Alcoólicas: Sob uma Perspectiva Bioecológica* [Transition for Emerging Adults and Alcohol Consumption: A Bioecological Perspective]. It adopts a qualitative, exploratory, and descriptive approach, guided by the Consolidated Criteria for Reporting Qualitative Research (COREQ), in accordance with the 32-item checklist^(14)^. This approach is appropriate given the study’s objective, as qualitative research aims to understand and describe complex and dynamic phenomena, thereby contributing to a deeper understanding of the topic under investigation. Moreover, it allows for the exploration of the diversity and richness of human experiences^(15)^.

### Study Context

This study was conducted at the Federal University of Rio Grande (FURG), located in the city of Rio Grande, RS, in the southernmost region of Brazil. The research settings included several university departments: the School of Medicine (FAMED) and the Undergraduate Program in Medicine; the Carreiros Campus, specifically the Undergraduate Program in Biology – Teacher Education Track, in the Institute of Biological Sciences; the Undergraduate Program in Civil Engineering, in the School of Engineering and the Undergraduate Program in Psychology, in the Institute of Human and Information Sciences.

### Participants and Selection Criteria

Participants were selected using a non-probabilistic sampling method. A total of 31 university students participated: 9 from the Undergraduate Program in Biology, 6 from Civil Engineering, 12 from Psychology, and 4 from Medicine. Of these, 23 identified as women and 8 as men. Data saturation was reached with this number of participants, as responses became repetitive and all research questions had been addressed. The inclusion criteria were: being between 18 and 24 years old; being regularly enrolled in one of the selected undergraduate programs (Biology, Medicine, Psychology, or Civil Engineering); and having consumed alcohol within the 12 months prior to data collection. This age range aligns with the sociocultural context of Brazil, where individuals aged 18 to 24—commonly referred to as young adults—are included in the definition of emerging adulthood^(16)^. Exclusion criteria included being unable to participate in the interview due to academic commitments, such as exams or tests, and failing to attend the scheduled interview on three consecutive occasions.

### Data Collection

Data were collected between September 17 and October 24, 2024, through semi-structured interviews. A digital form with a QR code was created to recruit participants. The form provided information about the study and asked respondents to indicate their availability and preferred location for the interview. It was disseminated via social media, primarily Instagram and WhatsApp. Interested students accessed the study details by scanning the QR code. In addition, university students were invited in person by the master’s student, who visited classrooms to present the study. A total of 57 students completed the form. The first two participants were selected to validate the semi-structured interview script. Nine students withdrew from the study, and 24 were excluded: 10 for being over 24 years old, 5 for not having consumed alcohol in the previous 12 months, 2 who declined participation after reading the informed consent form, and 7 due to scheduling conflicts. Interviews were conducted with the remaining 31 participants in private settings to ensure confidentiality and anonymity.

Before beginning the interviews, the master’s student (primary author) from the Graduate Program in Nursing (PPGEnf) introduced herself and explained the study’s objectives and methodology. Participants who agreed to take part were provided with two copies of the informed consent form—one to keep and one to return, signed, to the researcher. The interviews lasted between 26 and 70 minutes and were recorded using a mobile application downloaded from the Play Store. Participants were identified by the letter “P” followed by a number corresponding to the order of the interviews (e.g., P1 for the first participant, P2 for the second, and so on, up to P31). Additionally, a letter indicating the participant’s academic program was included: B for Biology, E for Civil Engineering, P for Psychology, and M for Medicine.

### Data Analysis and Processing

The data were analyzed using thematic content analysis, as proposed by Minayo. This method involves a set of communication analysis procedures aimed at systematically describing the content of messages to extract the information necessary for the study. It focuses on a specific area of knowledge and allows for the identification of key themes related to the research objectives.

Thematic content analysis consists of three phases: (a) pre-analysis, during which the collected material is read; (b) exploration of the material, when data analysis begins; and (c) processing and interpretation of results, when the data are discussed^(15)^. In the initial phase, the interviews were transcribed using Turbo Scribe software, compared to the audio recordings, and organized to form the final corpus. A comprehensive reading of the transcripts led to the development of initial categories based on constructs from Bronfenbrenner’s bioecological theory of human development. This theory explains human development as a result of dynamic interactions between the individual and various environmental systems over time. It has four central components: Process, Person, Context, and Time. In this study, emphasis was placed on the Person component, which refers to the individual characteristics—dispositions, resources, and demands—that each person brings to their experiences and that shape how they perceive, respond to, and interact with their environment^(17)^. This perspective was adopted based on the hypothesis that such individual characteristics directly influence young people’s perception and understanding of what constitutes excessive alcohol consumption, the threshold that defines it, and its health consequences. This approach enabled the analysis to capture the diversity of participants’ representations and knowledge, taking their individual characteristics into account. The categories developed from the data are directly linked to this theoretical component.

In the second phase, corresponding to the exploration of the material, participants’ reports were coded and grouped into categories using Atlas.ti software. Initially, the data were coded using the following labels: perception regarding alcohol consumption; knowledge about excessive alcohol use and consumption patterns that may affect biopsychosocial health; and knowledge of the negative impacts of excessive alcohol consumption. These initial codes were later reorganized into two analytical categories: Perception of Emerging Adults Regarding the Excessive Alcohol Use and Knowledge of Emerging Adults Regarding the Consequences of Excessive Alcohol Consumption for Biopsychosocial Health and Well-Being.

The third and final phase involved discussing the data based on the theoretical constructs and authors adopted in this study, particularly those related to the guiding framework. As part of the feedback process, participants received an invitation summarizing the study’s findings.

### Ethical Aspects

Regarding ethical considerations, this study was approved by the Institutional Review Board of the Health Department at FURG, under Opinion No. 7.033.705 and CAAE: 81929224.5.0000.5324. All procedures complied with the guidelines established in CNS Resolution No. 510/2016 for research involving human subjects. Authorization to conduct the study was also obtained from the coordinators of the aforementioned undergraduate programs.

## RESULTS

### Participant Characterization

Thirty-one students aged 18 to 24 participated in the study. Among them, 29.03% were 21 years old, 22.58% were 20, and 12.9% were 22, 23, or 19 years old, respectively. Additionally, 6.45% were 24, and 3.23% were 18 years old. Regarding gender, 23 participants identified as women and eight as men. All participants reported being single. In terms of living arrangements, nine lived with at least one family member (mother and/or father), four lived with a partner, and 16 lived alone. Twelve participants were from the city of Rio Grande, while 19 were from other municipalities, including: Vitória da Conquista (BA); Brasília (DF); Goiás Velho (GO); Alegrete, Bagé, Camaquã, Canguçu, Estrela, Gravataí, Pedro Osório, Pelotas, Santa Maria, Santana do Livramento, São Gabriel, Sarandi, Tavares (RS), and Piracicaba (SP).

### Emerging Adults’ Perceptions Regarding Excessive Alcohol Use

This category highlights college students’ perceptions of what constitutes excessive alcohol consumption and the patterns they associate with it. The analysis aimed to determine whether participants recognized when alcohol use becomes excessive and whether they were aware of the quantities considered harmful to health. According to participants PB1, PB3, PP4, PP7, PE8, PE10, PP11, PP14, PP17, PM20, PM21, PB23, PB25, PE27, and PE31, excessive use is characterized by daily or frequent (several times per week) alcohol consumption, as illustrated in the following excerpts:

*Every single day I go to a party, every single day I get drunk. I think that’s excessive use. I drink every day because I love beer—this is excessive for me. Nowadays, I think people drink more beer than water. So, I understand excessive use as drinking every day, even if it’s just a small amount ^(PB25)^.*

*I think it would be something that happens every day and at any time. For example, for me it is very surreal for someone to be drinking alcohol at 2 p.m. on a Wednesday, you know? So, from the moment someone starts drinking in the morning, afternoon, and evening, every day, with no control, for me that’s already excessive ^(PB3)^.*

According to PP5, PP6, PP7, PP9, PM20, PB23, and PM24, excessive use is associated with the underlying reasons for alcohol consumption—such as using it to relieve stress, escape from reality, or consuming it without self-control.

*I think it must be a way to escape reality, to forget all the problems^(PB23)^.*

*I think that’s drinking alcohol at times when you shouldn’t—for example, when you’re sad, so you don’t get depressed, or drinking every day just to relax ^(PM24)^.*

According to PE10, PP17, PP19, and PE27, excessive use occurs when a person feels unwell after consuming alcohol, experiencing unpleasant physical symptoms such as dizziness, nausea, and vomiting, or when they lose the ability to maintain self-control and ensure their own safety.

*I think that when you start to feel dizzy, sick, and can’t do things, that’s already harmful to your health^(PP17)^.*

*You keep drinking and drinking, and you start to feel sick, you know? You start to vomit. Or, like, you don’t know what’s happening anymore. You don’t know what you’re doing. You can’t even walk properly, and you keep drinking^(PP19)^.*

Regarding the standard dose that can affect health, participants PB1, PB3, PP4, PP6, PE8, PE10, PE15, PP16, PB18, PM20, PM22, PB25, and PE26 reported that daily alcohol consumption—regardless of the amount—can be harmful to health, as the cumulative effects over a lifetime may lead to chemical dependency.

*Maybe a bottle of wine, for example, or a couple of bottles of beer a week. I think that would already have some impact, to some extent ^(PP4)^.*

*Like, drinking a glass of beer every day is already a lot, you know? Once you get into that routine, a glass a day is no longer enough—then you start increasing the amount you drink each day^(PE8)^.*

Participants PP16, PB23, PM24, PB25, and PE26 reported that they do not know the amount of alcohol consumption that can be considered harmful to health.

*Quantity, I don’t know. But frequency—I think if you drink every week, twice a week is already too much ^(PM24)^.*

*I think that daily frequency can be harmful to your health. As for quantity, I’m not sure. What I do know is that frequent use can lead to alcoholism ^(PE26)^.*

Only participants PP5, PP9, PP14, PM20, and PM21 explicitly mentioned both frequency and quantity as patterns of alcohol consumption that may be harmful to health.

*I think that alcohol consumption, at least in larger quantities, once a week—once or twice a week in large quantities—or every day is already harmful ^(PP5)^.*

*I think there are two types of consumption: very frequent, even if in small quantities, and very intense consumption, even if infrequent. Of course, intense and frequent together is the worst-case scenario. But I think that even if you drink three times a month and get drunk to the point of losing consciousness and not remembering anything, that’s not healthy consumption. At the same time, drinking two or three glasses every day is also not healthy in the long term^(PP9)^.*

### Knowledge of Emerging Adults Regarding the Consequences of Excessive Alcohol Consumption on Biopsychosocial Health and Well-Being

This category presents the knowledge that emerging adult college students have about the effects of excessive alcohol consumption on biopsychosocial health and the present and future well-being of both the individuals who consume alcohol and those around them. Participants’ reports indicate that excessive consumption can compromise professional and social relationships, negatively affect physical and mental health, and lead to both immediate and long-term consequences. These effects may also place additional strain on the healthcare system and social services due to the resulting social and financial challenges.

Participants PB1, PB3, PP5, PP6, PP7, PP9, PE10, PP12, PP13, PP14, PP17, PB18, PM20, PM21, PM22, PB23, PM24, and PE27 reported that excessive alcohol consumption can have societal impacts, particularly due to the frequency of traffic accidents caused by intoxicated drivers and the increased incidence of chronic non-communicable diseases. These outcomes contribute to rising costs in healthcare and social services.

Overloading the SUS, for example, and the psychosocial care systems for people with substance use disorders, and consequently their families, right? I think it’s because treatment is collective—not just for the person who is addicted and using excessively, but for their entire context^(PP13)^.

*It causes more accidents, both in traffic, with people driving under the influence of alcohol, and also in cases of domestic violence, deaths, and even street fights^(PB23)^.*

Changes in behavior can negatively impact family, friendship, and professional relationships due to aggression, impulsiveness, or inappropriate conduct. Emotional and physical absence may lead to the distancing of close contacts. As a result, deterioration of one’s social and professional image becomes evident, often accompanied by impaired daily functioning and difficulty in fulfilling responsibilities.

*When people are like this, they spend most of their time upset, and they also miss out on a lot of things. They miss out on family time, on things related to emotional well-being^(PE10)^.*

*They lose their sense of modesty, you know? I don’t know. They lose their sense of respect for other people’s space. I think it makes others uncomfortable^(PP11)^.*

Continuous alcohol consumption can compromise the financial situation of individuals who drink excessively, as a significant portion of their income may be spent on alcohol, undermining their ability to meet basic needs. It can also interfere with the pursuit of personal and professional goals, while reducing productivity and performance in daily activities.

*Spending money on alcohol affects people a lot, doesn’t it? It ends up affecting their health. They spend money on alcohol and then have nothing to eat^(PB23)^.*

*The person may not be able to focus on what they need to do because they become very dependent on alcohol. They constantly want to drink, so they can’t concentrate on anything else, like studying or exercising^(PM24)^.*

Participants PB1, PB3, PP4, PP6, PE8, PP12, PP13, PE15, PP16, PP19, PM21, PB23, PM24, PB25, PE26, PE27, and PB28 reported that excessive alcohol consumption can cause long-term harm to an individual’s health due to the associated risks, including liver, heart, neurological, and kidney problems, increased susceptibility to various types of cancer, alcoholic coma, and premature death. Another significant concern is the impaired ability to build future life projects or achieve meaningful personal goals. These factors ultimately limit well-being and compromise the possibility of leading a healthy life.

*It doesn’t only affect the person while they’re drinking, right? For example, with cirrhosis—if the person stops drinking for a while, they’ll still feel the effects in the future. Or they may end up being predisposed to other diseases because of the period when they drank too much^(PP4)^.*

*Excessive alcohol consumption can stress people out. It can lead to more fragile, conflicting, and distant relationships, and even long-term breakups^(PP6)^.*

Most of the time, the learning process is impaired, as individuals who consume excessive amounts of alcohol are often unable to perform daily activities and may reach the extreme of losing their personal and professional functionality altogether.

*The prospects for a future life are harmed. I think the person loses some sense of direction—of where they want to go. Maybe they get stuck in that world of just partying, drinking, and everything else, and end up losing sight of their goals. Or sometimes, they’re people who already don’t know where they want to go, and because of that, they end up turning to alcohol, and drinking becomes a kind of comfort for their suffering^(PE10)^.*

Regarding mental health, participants PB1, PP6, PP7, PE8, PP9, PP14, PE15, PB18, PM21, PM22, PM24, PB25, PB28, PB29, and PB30 reported that excessive alcohol consumption can affect brain structures, potentially leading to a predisposition to various mental disorders, such as depression, anxiety, intense stress, and chemical dependency.

*Any problem, anything, you’ll try to seek that momentary happiness, and each time you’ll increase the dose to try to fill what’s missing in your life. Like any addiction—it starts small, and you end up needing to consume more to get the same effect^(PB28)^.*

Participants also viewed excessive alcohol consumption as a major factor contributing to the decline in a person’s future quality of life, particularly in old age.

*I think that drinking too much will definitely reduce a person’s life expectancy. They’ll grow older with more health problems, and with less mobility and independence^(PE15)^.*

*When a person gets older, they’ll start to notice the effects of their lifestyle. They may feel that their quality of life has significantly declined with age^(PM21)^.*

## DISCUSSION

Despite the diversity of participants, the results of this study revealed that all had difficulty understanding the concept of excessive alcohol consumption and identifying the pattern considered excessive. According to the National Institute on Alcohol Abuse and Alcoholism (NIAAA), excessive alcohol consumption refers to intake that exceeds moderate levels, increasing the risk of health problems and other negative outcomes. This definition includes specific categories, such as binge drinking—defined as consuming five or more alcoholic drinks for men, or four or more for women, within approximately two hours—and heavy drinking, which refers to the weekly intake of 15 or more drinks for men and 8 or more for women^(18)^. In Brazil, there is no officially established standard drink definition. However, the *Centro de Informação sobre Saúde e* Álcool *(CISA)* [Center for Information on Health and Alcohol] adopts the NIAAA criteria, which defines a standard drink as containing 14 grams of pure alcohol. This corresponds to approximately 355 ml of beer (5% alcohol content), 150 ml of wine (12%), or 45 ml of distilled spirits such as vodka, whiskey, cachaça, gin, or tequila (40%)^(11)^.

However, the study participants tended to interpret binge drinking primarily in terms of frequency, rather than the quantity consumed within a short period. This understanding reveals a gap in their knowledge about the specific risks associated with rapid and high alcohol intake, even when such episodes do not occur daily. Additionally, although the study did not use a standardized measurement scale, participants’ responses suggest that they are unaware of the exact amount that constitutes a single serving of alcohol and its potential harm to health. This lack of knowledge can have important consequences for emerging adult college students. Without clear reference points, they may unknowingly consume excessive amounts, increasing the risk of alcohol poisoning and engagement in risky behaviors. In the university context—where alcohol consumption is often shaped by social dynamics—this lack of information may lead students to rely on social norms, rather than evidence-based guidelines, to gauge how much they are drinking.

The Bioecological Theory of Human Development—particularly the Person component, which comprises three interdependent dimensions (dispositions, resources, and biological characteristics)—provided a valuable framework for interpreting the results. Dispositions refer to internal tendencies such as motivation, curiosity, and behavioral choices, including those related to alcohol consumption. In this context, there is a tendency to normalize excessive alcohol use over short periods, especially in social environments where heavy drinking is encouraged and socially reinforced. A lack of knowledge about the potential harms of alcohol weakens the individual’s capacity for self-regulation and increases vulnerability to external pressures. These dispositions contribute to a form of psychological tolerance that diminishes receptiveness to prevention messages and limits critical reflection on personal behavior.

Regarding resources, Bronfenbrenner defines them as the knowledge, cognitive abilities, and skills that guide individuals in making choices within their environment. In this sense, the lack of a clear perception of risk and the fragmented understanding of the effects of alcohol consumption reflect a deficiency in specific information—information that is essential for self-regulation and effective prevention among emerging adult college students. This gap contributes to a distorted assessment of one’s own behavior and results in limited understanding of the mechanisms that link excessive consumption to serious biopsychosocial consequences, including transmissible infections and resistance to medical treatments.

Biological characteristics, in turn, refer to physiological, genetic, and developmental factors that influence individual responses to alcohol. The scientific literature establishes different consumption limits based on gender, as women are more vulnerable to the effects of alcohol due to metabolic and physical differences^(19,20)^. Despite this, none of the 23 women who participated in the study mentioned this vulnerability in their responses, which likely indicates a lack of awareness about gender-specific risks. Considering that emerging adult college students are in a transitional phase between adolescence and adulthood—a period during which the brain is still developing—excessive alcohol consumption may compromise neurological and cognitive functions^(21,22)^. This aspect was also absent from participants’ responses, further reinforcing the limited understanding of how biological characteristics influence alcohol consumption and its consequences.

Regarding the risks to health and biopsychosocial well-being, participants identified some short-term consequences, such as fatigue, regret, and changes in social interactions. While long-term effects were mentioned, they were limited to liver and kidney diseases, traffic accidents, and family conflicts. The analysis indicates a partial understanding of the impacts of excessive alcohol consumption, marked by limited information and a lack of connection between causes, mechanisms, and outcomes. This fragmented knowledge does not fully account for the social, economic, and public health harms associated with alcohol abuse, nor does it reflect awareness of the barriers it poses to achieving the Sustainable Development Goals.

The analysis of dispositions reveals that motivation, curiosity, and choices related to alcohol consumption are strongly influenced by social contexts, including family. By focusing primarily on frequency, participants overlook the risks associated with high intake over a short period, showing limited awareness of both the immediate and cumulative effects. As university students, they may be more susceptible to excessive consumption patterns^(8)^, underscoring the need for targeted educational interventions. Academic-related stress and emotional challenges—such as sadness, anxiety, and depression—are common in this population. In the absence of sufficient internal and external resources to support resilience, alcohol may be used as a coping strategy to alleviate emotional discomfort^(7)^. Although participants’ reports reflected this reality, they lacked the depth needed to fully recognize the underlying mechanisms of vulnerability and their long-term consequences.

Finally, considering that most of the emerging adult university students in this study are preparing for careers in the health and education fields, it is essential that they develop a scientific and comprehensive understanding of the harmful effects of alcohol abuse. This understanding must go beyond empirical knowledge and encompass biopsychosocial dimensions, individual and contextual risk factors, and the broader implications for public health. Such knowledge is fundamental for enabling these future professionals to actively promote both their own health and the health of the communities they serve.

Some researchers have examined emerging adults’ knowledge of alcohol-related risks and standard drink units in specific quantities of beer, wine, and spirits. Among college students who consume these beverages, attitudes and practices often occur without a clear understanding of what constitutes excessive consumption. The findings of this study confirm that alcohol use is a common behavior among emerging adult university students. However, even those enrolled in health-related programs demonstrated limited knowledge about the harmful effects of alcohol on health and well-being. A study conducted in South Africa with undergraduate students, which aimed to assess knowledge, attitudes, and practices related to alcohol consumption, found that approximately one-third of participants did not respond to questions about standard drink units for different types of beverages such as beer, wine, and spirits. Among those who did respond, 71.3% underestimated the amount corresponding to a standard unit^(23)^. Similarly, a cross-sectional study conducted in Poland with 480 university students aged 18 to 35 identified a low level of knowledge regarding the health effects of alcohol consumption^(24)^.

A study conducted in Portugal with emerging adult university students found that, overall, participants demonstrated low levels of knowledge regarding the negative consequences of alcohol on health and well-being. Interestingly, those with higher knowledge scores reported greater alcohol consumption compared to those with lower scores^(25)^. This may reflect a lack of understanding about the specific consumption limits that pose health risks. Similarly, a study conducted in Italy with 1,928 university students aged 18 to 24 revealed that participants were not fully aware of the harmful effects of excessive alcohol consumption^(26)^. A study conducted in China reported similar findings^(27)^.

Most studies on alcohol consumption are quantitative in nature and focus on the general population. In contrast, one of the distinguishing features of the present research lies in its qualitative approach, which allowed emerging adult college students to reflect on their personal histories and examine the construction of concepts and paradigms related to alcohol use. This approach also encouraged them to reconsider the repercussions of their current consumption on their academic and professional trajectories. Another unique aspect of this study is its focus on a specific population—emerging adult university students. The findings underscore the value of this type of research, as it enabled a deeper understanding of participants’ perceptions of risk, patterns of excessive consumption, and the impact on their biopsychosocial health and well-being. Moreover, it prompted participants to reflect on how their drinking behaviors were already affecting their academic performance and how, if continued, these patterns could jeopardize future professional opportunities.

## CONCLUSION

The results revealed that participants lack in-depth knowledge about the negative repercussions and risks of excessive alcohol consumption on biopsychosocial health and well-being. They were unaware of the number of drinks considered standard for defining excessive use, the fact that the brain is still developing during this life stage, and the relationship between gender and alcohol-related vulnerability.

As a contribution to the health sector, the results of this study may support the planning of initiatives focused on raising awareness and promoting health education about the harms of excessive alcohol consumption to health and quality of life. These actions should address key topics such as: the definition of excessive consumption; what constitutes a standard drink and the corresponding amount of alcohol (in milliliters) depending on the type of beverage; gender-related differences in alcohol effects; socioeconomic and health consequences; signs of alcohol dependence; and the physiological reasons why alcohol is contraindicated for minors. These aspects are particularly relevant for the emerging adult university student population.

In addition to the need for health awareness and education campaigns, it is essential to support emerging adult college students in developing healthy strategies for managing time and stress, in order to protect their mental health. When conducting such campaigns, health professionals should emphasize both the rationale for and the methods of controlling alcohol consumption. Presenting real-life examples of the medium- and long-term negative consequences of alcohol use on biopsychosocial health and quality of life may help students better understand the purpose of warnings and restrictions. This approach reinforces the idea that such guidance is not meant to restrict their freedom or enjoyment, but to prevent harm to their own well-being and that of those around them.

Limitations of this study include the predominance of female participants, who may generally have different perspectives on alcohol consumption compared to men. Additionally, the data were collected within a specific sociocultural context, which may have influenced participants’ perceptions of what constitutes excessive alcohol use. The study was conducted at a single higher education institution and involved a small number of participants, as it followed a qualitative approach that prioritized students’ experiences and subjectivities.

Finally, the gaps identified in this study, along with those previously discussed, open new possibilities for future research on alcohol consumption among emerging adult university students. These findings also invite reflection on the role of public policies related to alcohol and other drug use, as well as the broader social responsibility of universities in promoting a better quality of life for this specific population—one that largely represents the productive workforce of a country.

## Data Availability

The data underlying this study have been deposited in the SciELO Data repository, which can be accessed through the following electronic address https://data.scielo.org/dataset.xhtml?persistentId=doi:10.48331/SCIELODATA.RU7N8N.
