# Supplementary material for: Knowledge of the consequences of excessive alcohol consumption among emerging adult college students
Source: Rev Esc Enferm USP. 2025 Aug 22;59:e20250088. doi: 10.1590/1980-220X-REEUSP-2025-0088en (PMC12373133; doi:10.1590/1980-220X-REEUSP-2025-0088en)
Supplement: Translation

# Translation: Adultos emergentes universitários e o conhecimento relativo às consequências do consumo excessivo de bebidas alcoólicas

**DOI:** 10.1590/1980-220X-REEUSP-2025-0088pt

**Authors:** Roselande Simon, Adriane Maria Netto de Oliveira, Cristiane Barros Marcos, Mara Regina Santos da Silva, Dominique Therrien, Patrícia Bitencourt Toscani Greco, Evy Nazon, Mara Regina Pombo Amaral, Émilen Vieira Simões

**Affiliations:** 1 Universidade Federal do Rio Grande, Programa de Pós-Graduação em Enfermagem, Rio Grande, RS, Brasil.; 2 Universidade Federal do Rio Grande, Instituto de Ciências Biológicas, Rio Grande, RS, Brasil.; 3 Université du Québec en Outaouais, Département des Sciences Infirmières, Gatineau, Québec, Canadá.

**Keywords:** Consumo de Bebidas Alcoólicas, Estudantes, Saúde Humana, Bem-Estar Psicológico

## Abstract

**Objetivo:**

Compreender a percepção e o conhecimento dos adultos emergentes universitários sobre as consequências do consumo excessivo de bebidas alcoólicas para a saúde biopsicossocial, bem-estar atual e futuro das pessoas.

**Método:**

Trata-se de um estudo exploratório, descritivo e de abordagem qualitativa, realizado com 31 estudantes universitários. Um formulário com código QR foi divulgado online para informar sobre a pesquisa. A coleta de dados foi conduzida por meio de entrevista semiestruturada. Os dados foram transcritos por meio do software Turbo Scribe e os discursos analisados com o auxílio do software Atlas.ti, utilizando a técnica de análise de conteúdo.

**Resultados:**

Existe uma lacuna no conhecimento dos participantes sobre o que é o consumo excessivo de bebidas alcoólicas e as suas repercussões para a saúde. Conclui-se que é fundamental promover campanhas contínuas de conscientização e educação em saúde, a fim de ampliar o conhecimento e favorecer decisões conscientes quanto ao consumo de álcool.

## INTRODUÇÃO

O álcool é a substância psicoativa mais consumida no mundo por meio de bebidas alcoólicas. O consumo pode ser moderado, excessivo ou nocivo e ocasionar a dependência alcoólica. De acordo com a Organização Mundial da Saúde (OMS), todo consumo de álcool é nocivo para a saúde e para a vida das pessoas^(1)^. Estudos mostram que o uso de bebidas alcoólicas contribui para o aumento global da prevalência de doenças não transmissíveis, acidentes de trânsito, violência doméstica, violência física, violência psicológica, abuso sexual e aumento da mortalidade. Isso eleva os gastos com assistência sanitária, custos sociais e legais^(2,3)^. Por estes motivos, ele é considerado um grave problema de saúde pública e uma das causas de morte evitável.

Em todas as faixas etárias, os adultos emergentes são aqueles com uma taxa de consumo mais alta e de abstinência mais baixa^(4,5)^. Adulto emergente é um conceito definido pelo psicólogo americano Jeffrey Jensen Arnet em 2000, como sendo as características específicas do desenvolvimento de jovens com idade entre 18 e 25 anos. As cinco características específicas são: a exploração da identidade, período durante o qual o jovem está buscando a resposta para a pergunta quem sou eu, seja em termos pessoais, relacionais e/ou profissionais. A segunda, refere-se à instabilidade, ela é explicada pelo fato de que, durante essa fase, os jovens ainda não atingiram a estabilidade típica da vida adulta. Frequentemente, estão em processo de exploração profissional, mudam de residência, e suas metas e relacionamentos tendem a se transformar diversas vezes, até que consigam definir com mais clareza o que desejam para si. A terceira característica é definida como a idade que exige ainda mais responsabilidades, gerando, muitas vezes, uma sobrecarga de vida, pois este é o período no qual são estabelecidas as bases para a qualidade de vida na fase adulta. A quarta característica está relacionada ao sentimento de ambivalência, o qual mostra que esses jovens não são mais adolescentes, mas também ainda não são adultos. E, a quinta característica se refere a uma idade de muitas possibilidades, portanto, esse período é marcado por grandes oportunidades de preparação para uma boa qualidade de vida durante o ciclo vital^(6)^.

Na população de adultos emergentes, a literatura mostra que aqueles que frequentam a universidade têm maior prevalência de consumo de bebidas alcoólicas em relação aos jovens da mesma idade que não são estudantes universitários. Provavelmente isto ocorre porque eles participam de atividades ou eventos que facilitam o consumo excessivo de álcool, como festas, jogos e atividades de lazer entre estudantes universitários^(7,8)^. A aquisição do conhecimento relativo às consequências negativas do consumo excessivo de álcool e do padrão considerado moderado, poderá permitir o uso moderado da substância psicoativa, estimulando os adultos emergentes universitários a adotarem hábitos saudáveis na vida cotidiana. Outro aspecto refere-se aos fatores de risco que, ao serem reconhecidos permitem a tomada de decisões de forma consciente^(9)^.

Informações relacionadas as consequências negativas do consumo excessivo de bebidas alcoólicas, bem como o padrão que o define, pode ajudar os adultos emergentes a definirem se este comportamento vale a pena, mesmo comparado aos riscos envolvidos. Provavelmente, o conhecimento facilita as pessoas priorizarem ações mais saudáveis em seu cotidiano, a fim de manter a saúde biopsicossocial e bem-estar, tanto próprio quanto o da sua família e comunidade^(10)^.

Estudos mostram que o pares exercem grande influência no consumo de bebidas alcoólicas pelos adultos emergentes universitários^(7,8)^. Assim, quando se relacionam com estes fazem uso moderado e, tendem a adotar e manter o mesmo comportamento.

De acordo com o último relatório da OMS o consumo de bebidas alcoólicas em 2019, foi responsável por cerca de 2,6 milhões de mortes. Adicionalmente, 7% da população mundial com 15 anos e mais tem, no mínimo, um transtorno mental relacionado ao consumo de álcool, sendo o mais prevalente, a dependência química, com uma taxa de 3,7%^(1)^. No Brasil, 50 mil casos de internação foram registrados no ano de 2023, a taxa de óbitos atribuíveis ao álcool por100 mil habitantes foi de 33%, em 2022^(11)^.

No Brasil, em 2019, os custos sociais com o consumo de álcool foram de 18,8 bilhões de reais^(12)^ Em função dessas evidências, provavelmente promover o conhecimento relativo as consequências negativas do consumo excessivo e a quantidade de unidades que o definem seja um investimento relevante e sustentável. A partir dessas informações e conscientização, diversas autoridades da saúde, educacionais, políticas, sociais, da segurança pública, dentre outras, poderão direcionar ações para a implementação de campanhas que construam bases sólidas para incentivar o consumo moderado. Essas ações podem contribuir positivamente para a saúde biopsicossocial e o bem-estar geral dos indivíduos, incluindo-se aí os adultos emergentes universitários.

De acordo com as simulações dos países das Organizações para Cooperação e Desenvolvimento Econômico (OCDE), os custos socioeconômicos do consumo de álcool entre 2020 a 2050, podem chegar a $138 bilhões US por ano, somados a uma perda de aproximadamente 33 milhões de trabalhadores e uma queda de 1,6% do PIB^(13)^. Tais dados justificam a realização do presente estudo. Por outro lado, embora o consumo de bebidas alcoólicas entre jovens universitários já tenha sido amplamente estudado, poucos trabalhos se dedicaram a examinar o conhecimento dos adultos emergentes universitários sobre o que constitui um consumo excessivo, bem como sobre as implicações desse consumo para a saúde e o bem-estar, tanto no presente quanto no futuro. Por isso, o objetivo deste artigo é compreender a percepção e o conhecimento dos adultos emergentes universitários sobre as consequências do consumo excessivo de bebidas alcoólicas para a saúde biopsicossocial, bem-estar atual e futuro das pessoas.

## MÉTODO

### Tipo de Estudo

Este estudo é um recorte da dissertação de mestrado intitulada: “Transição para adultos emergentes e o consumo de Bebidas alcóolicas: Sob Uma Perspectiva Bioecológica”. Trata-se de um estudo com abordagem qualitativa, exploratória e descritiva guiada pelas diretrizes da Consolidated Criteria for Reporting Qualitative Research (COREQ), obedecendo aos 32 itens estabelecidos pelo checklist^(14)^. A escolha por essa abordagem justifica-se por seu objetivo, uma vez que tal tipo de pesquisa tem seu foco na compreensão e descrição dos problemas e fenômenos complexos e dinâmicos, contribuindo para uma melhor apreensão do objeto de estudo. Além disso, possibilita aos pesquisadores compreenderem a diversidade e a riqueza das experiências humanas^(15)^.

### Contextos do Estudo

O estudo foi realizado na Universidade Federal do Rio Grande (FURG), situada no município do Rio Grande/RS, no extremo sul do Brasil. Os contextos investigativos foram a Unidade de Saúde da Área Acadêmica, e em específico: a Faculdade de Medicina (FAMED) e o Curso de Graduação em Medicina; o Campus Carreiros, Instituto de Ciências Biológicas (ICB), especificamente o Curso de Graduação em Biologia Licenciatura; a Escola de Engenharia (EE-FURG), especificamente o Curso de Graduação em Engenharia Civil e finalmente o Instituto de Ciências Humanas e da Informação (ICHI), especificamente, o Curso de Graduação em Psicologia.

### Participantes e Critérios de Seleção

Os participantes foram selecionados por meio de um método de amostragem não probabilística. No total, participaram 31 estudantes universitários: 9 do Curso de Graduação em Biologia Licenciatura; 6 do Curso de Graduação em Engenharia Civil; 12 do Curso de Graduação em Psicologia e 4 do Curso de Graduação em Medicina. Em relação ao gênero, 23 participantes se identificaram como mulheres e 8 como homens. A saturação dos dados foi alcançada com esse número, uma vez que as respostas obtidas se tornaram repetitivas e todas as questões da pesquisa já haviam sido contempladas. Foram adotados como critérios de inclusão: o(a) estudante ter entre 18 e 24 anos; estar regularmente matriculado no Curso de Graduação em Biologia Licenciatura, Medicina, Psicologia e Engenharia Civil e, fazerem uso ou terem consumido bebidas alcoólicas nos últimos doze meses até o momento da coleta de dados. Esta faixa etária faz parte de um processo de adaptação ao contexto sociocultural do Brasil, o qual determina que entre as idades mencionadas neste período da vida, o grupo com idade entre os 18 e os 24 anos, comumente conhecidos como jovens-jovens encontram-se inseridos no referencial utilizado sobre os adultos emergentes^(16)^. Os critérios de exclusão foram: os estudantes que não puderam participar da entrevista devido à realização das provas, dos exames ou em função da realização de outras atividades acadêmicas e, aqueles que não compareceram à entrevista por três vezes consecutivas.

### Coleta dos Dados

Os dados foram coletados de 17.09.2024 a 24.10.2024, através de uma entrevista semiestruturada. Para entrar em contato com os estudantes universitários foi preparado um formulário com um código QR, explicando os objetivos da pesquisa, solicitando o local e melhor horário para realização da entrevista com os participantes. Os formulários foram compartilhados via redes sociais, principalmente pelo Instagram e whatsapp. Aqueles que quiseram participar escaneavam o código QR, para ter acesso às informações da pesquisa. Outro modo de solicitar a participação dos estudantes universitários foi por meio presencial, quando a mestranda foi nas salas de aula falar a respeito do estudo e incentivá-los. No total, 57 estudantes preencheram o formulário. Desses, os dois primeiros participantes foram selecionados para validar o roteiro da entrevista semiestruturada. Nove desistiram, além disso, 24 foram excluídos da amostra: 10 por terem mais de 24 anos, 5 por não terem consumido álcool nos últimos 12 meses, 02 desistiram após a leitura do TCLE e 07 por não terem tempo para participar da entrevista. As entrevistas foram realizadas com 31 participantes, em local reservado, garantindo a privacidade e anonimato dos participantes.

Antes de iniciar as entrevistas, a mestranda (Primeira autora) do Programa de Pós Graduação da Enfermagem (PPGEnf) se apresentou e explicou os objetivos e a metodologia da pesquisa. Logo após, para aqueles que concordaram em participar, foi dado o Termo de Consentimento Livre e Esclarecido (TCLE) em duas vias para assinar, ficando uma cópia com ele e a outra com a mestranda. As entrevistas tiveram duração entre 26 e 70 minutos e, foram gravadas usando o aplicativo do *play store*. Os participantes foram identificados pela letra “P”, a qual foi numerada, à medida que as entrevistas iam sendo realizadas, ou seja, o primeiro participante foi registrado como número P1, o segundo como P2, e assim sucessivamente até o participante 31, seguido da primeira letra do curso, sendo B para Biologia, E para Engenharia Civil, P para Psicologia e M para Medicina.

### Analise e Tratamento dos Dados

Os dados foram analisados por meio da análise de conteúdo temática de Minayo. Trata-se de uma combinação de procedimentos de análise da comunicação, com a ideia de descrever o conteúdo das mensagens por meio de etapas sistemáticas, para obter as informações necessárias para realização da pesquisa. Este tipo de análise considera um tema específico do conhecimento.

A análise temática é composta por três fases: (a) pré-análise, quando é realizada a leitura do material coletado; (b) exploração do material, período em que é iniciada a análise dos dados; (c) tratamento e interpretação dos resultados, quando realiza-se a discussão dos dados^(15)^. Na etapa inicial, as entrevistas foram transcritas com o auxílio do software Turbo Scribe, revisadas com base nos áudios, e organizadas para formar o corpus final. A partir da leitura compreensiva dos discursos, foram elaboradas categorias iniciais com base em constructos da teoria bioecológica do desenvolvimento humano de Bronfenbrenner. Essa teoria propõe uma compreensão do desenvolvimento humano com base nas interações dinâmicas entre o indivíduo e vários sistemas ambientais ao longo do tempo. Ela tem quatro componentes centrais: Processo, Pessoa, Contexto e Tempo. Neste trabalho, a ênfase está no componente pessoa, que se refere às características individuais (disposições, recursos e demandas) que cada indivíduo traz para suas experiências no mundo e que influenciam a maneira como percebe, responde e se adapta ao ambiente^(17)^. A adoção dessa perspectiva baseia-se na hipótese de que essas características influenciam diretamente a percepção e o entendimento dos jovens sobre o que é o consumo excessivo de álcool, a dose considerada excessiva e as consequências para a saúde. Essa abordagem possibilitou destacar a diversidade das representações e do conhecimento dos participantes, levando em conta suas características individuais. As categorias construídas a partir da análise de dados estão diretamente ligadas a esse componente.

Na segunda fase, que corresponde a exploração do material, os discursos dos participantes foram agrupados por categorias, utilizando o software Atlas.ti. Inicialmente, os dados foram categorizados com os seguintes códigos: percepção relativa ao consumo; conhecimento sobre o uso excessivo e o padrão de consumo que pode afetar a saúde biopsicossocial; conhecimento relativo aos impactos negativos do consumo excessivo. Posteriormente, as categorias foram novamente reagrupadas, da seguinte forma: Percepção dos Adultos Emergentes Relativa ao Uso Excessivo de Bebidas Alcoólicas; Conhecimento dos Adultos Emergentes Relativo às Consequências do Consumo Excessivo para a Saúde Biopsicossocial e Bem-Estar.

Naterceira e última fase foi realizada a discussão dos dados, a partir de conceitos e autores que trabalham com o tema proposto neste estudo, assim como utilizando os constructos da teoria usada. Aos participantes foi enviado um convite como feedback para informar os resultados do estudo realizado. O Zotero foi utilizado como gerenciador de referências.

### Aspectos Éticos

Em relação aos aspectos éticos da pesquisa, o estudo foi aprovado pelo Comitê de Ética em Pesquisa da Universidade Federal do Rio Grande (CEP/FURG)sob o parecer número nº 7.033.705 e CAAE: 81929224.5.0000.5324, seguindo todas as recomendações da Resolução CNS nº 510/2016 para pesquisas com seres humanos. A realização do estudo também foi autorizada pelos(as) coordenadores(as) dos Cursos de Graduação mencionados anteriormente.

## RESULTADOS

### Caracterização dos Participantes

Participaram do estudo trinta e um estudantes na faixa etária entre 18 e 24 anos. Destes, 29,03%, têm vinte e um anos, 22,58% têm vinte anos, 12, 9% têm vinte e dois anos, 12, 9% têm vinte e três anos, 12, 9%, têm dezenove anos, 6,45% têm vinte e quatro anos e 3,23% tem dezoito anos. No que se refere ao gênero, 23 se identificaram como mulheres e oito como homens. Todos se declararam solteiros. Nove estão morando com familiares (mãe e/ou pai), quatro moram com a namorada/namorado e, dezesseis moram sozinhos. Doze são da cidade do Rio Grande, enquanto dezenove pertencem a outras localidades, de acordo com as cidades e estados, a seguir: Vitória da Conquista (BA); Brasília (DF); Goiás Velho (GO); Alegrete (RS), Bagé (RS), Camaquã (RS), Canguçu (RS), Estrela (RS), Gravataí (RS), Pedro Osório (RS), Pelotas (RS), Santa Maria (RS), Santana do Livramento (RS), São Gabriel (RS), Sarandi (RS), Tavares (RS) e Piracicaba (SP).

### Percepção dos Adultos Emergentes Relativa ao uso Excessivo de Bebidas Alcoólicas

Nesta categoria foi dado destaque a percepção dos adultos emergentes universitários sobre a noção de consumo excessivo e o padrão que consideram como excessivo, o que permitiu entender se eles conhecem quando um consumo é considerado desta forma e se sabem a quantidade considerada prejudicial à saúde. De acordo com os PB1, PB3, PP4, PP7, PE8, PE10, PP11, PP14, PP17, PM20, PM21, PB23, PB25, PE27 e PE31 o uso excessivo é quando a pessoa consome bebidas alcoólicas diariamente ou várias vezes por semana, conforme descrito abaixo;

*Todo santo dia eu vou numa festa, todo santo dia eu fico bêbada, para mim é um uso excessivo. Todo santo dia eu bebo, porque eu amo cerveja, isto é excessivo para mim. Hoje mesmo eu acho que o pessoal bebe muito mais cerveja do que água. Então, eu entendo o uso excessivo como todo santo dia ou alguma quantidade diariamente, por mais que seja mínima* ^(PB25)^.

*Acho que seria aquela coisa de todo dia e em qualquer horário. Por exemplo, para mim é muito surreal alguém estar bebendo álcool 2 horas da tarde em plena quarta-feira, sabe? Então, a partir do momento que a pessoa passa a beber manhã, tarde e noite, todos os dias, o uso descontrolado, para mim já é excessivo* ^(PB3)^.

Na visão dos PP5, PP6, PP7, PP9, PM20, PB23 e PM24, o uso excessivo está relacionado ao motivo pelo qual a pessoa usa bebidas alcoólicas, seja para aliviar o estresse, fugir da realidade da vida e consumir sem autocontrole.

*Eu acho que deve ser uma forma de escapar da realidade. Para esquecer todos os problemas* ^(PB23)^.

*Eu acho que é isso, utilizar a bebida alcoólica em momentos que você não deveria, por exemplo, quando você está triste, daí não fica depressivo ou beber todos os dias para ficar relaxado* ^(PM24)^.

Na visão dos PE10, PP17, PP19, PE27, o uso excessivo é quando a pessoa sente mal-estar depois de consumir bebidas alcoólicas, ou seja, apresenta sintomas físicos desagradáveis como: tontura, náuseas e vômitos, ou quando se torna incapaz de manter o autocontrole e de se proteger.

*Acho que na medida que você já começa a se sentir tonto, ficar mal, não consegue fazer as coisas, eu acho que isso para mim já prejudica a saúde também* ^(PP17)^.

*Tu continuas bebendo, bebendo e começa a passar mal, sabe? Começa a vomitar. Ou, tipo, não sabe mais o que está acontecendo. Não sabe o que está fazendo. Não consegue nem caminhar direito e continua bebendo* ^(PP19)^.

No que diz respeito à dose padrão que pode afetar a saúde, os participantes PB1, PB3, PP4, PP6, PE8, PE10, PE15, PP16, PB18, PM20, PM22, PB25 e PE26 referem que o consumo diário, independentemente da quantidade ingerida, pode ser prejudicial à saúde, porque ao longo da vida, os efeitos cumulativos do consumo vão desenvolver a dependência química.

*Talvez uma garrafa de vinho, por exemplo. Ou umas duas garrafas de cerveja por semana. Acho que isso já causaria algum impacto, em alguma medida* ^(PP4)^.

*Tipo, beber um copo de cerveja todos os dias já é muito, entendeu? Depois que tu tens essa rotina, um copo por dia já não é mais nada, aí começa a aumentar a quantidade de bebida nos dias* ^(PE8)^.

Os participantes PP16, PB23, PM24, PB25, PE26, relataram que desconhecem a quantidade do consumo de bebidas alcoólicas que pode ser prejudicial à saúde.

*Quantidade, não sei. Mas frequência, eu acho que se você beber toda semana, duas vezes na semana é muito* ^(PM24)^.

*Eu acho que a frequência diária pode prejudicar a saúde. E a quantidade, não sei. Eu sei da questão de frequência, porque daí entra uma situação de alcoolismo* ^(PE26)^.

Somente os participantes PP5, PP9, PP14, PM20 e PM21mencionaram explicitamente tanto a frequência quanto a quantidade como um consumo ou padrão de uso que pode ser prejudicial à saúde.

*Eu acho que consumo de álcool, pelo menos em quantidades maiores, uma vez por semana. Uma, duas vezes por semana em grande quantidade, ou todos os dias, já é prejudicial* ^(PP5)^.

Eu acho que tem dois tipos de consumo, um muito frequente, mesmo que seja pouca quantidade, mas muito frequente e, um consumo muito intenso, mas que não seja frequente. Claro, um intenso e frequente acho que é o pior cenário. Mas eu acho que mesmo que seja três vezes no mês, tu tomar um porre, de ficar inconsciente, de não lembrar de nada, já acho que não é um consumo saudável, mas também todos os dias, tu tomar duas, três taças de alguma bebida, também acho que não é um consumo saudável a longo prazo^(PP9)^.

### Conhecimento dos Adultos Emergentes Relativo às Consequências do Consumo Excessivo para a Saúde Biopsicossocial e Bem-Estar

Nesta categoria são elencadas as informações ou conhecimentos que os adultos emergentes universitários possuem sobre os efeitos que o consumo excessivo de álcool tem sobre a saúde biopsicossocial e o bem-estar atual e futuro do consumidor e das pessoas em seu entorno. Os discursos dos estudantes universitários mostram que o consumo excessivo pode comprometer as relações profissionais e sociais, bem como afetar a saúde física e mental dos usuários com consequências negativas imediatas e ao longo da vida, inclusive sobrecarregar o sistema de saúde e os serviços sociais em função dos problemas sociais e financeiros.

Os participantes PB1, PB3, PP5, PP6, PP7, PP9, PE10, PP12, PP13, PP14, PP17, PB18, PM20, PM21, PM22, PB23, PM24 e PE27, relatam que o consumo excessivo pode afetar a sociedade, em função dos acidentes de trânsito frequentes, ocasionados por motoristas alcoolizados e das doenças crônicas não transmissíveis, com aumento dos custos com os serviços de saúde e serviços sociais.

*Sobrecarga do SUS, por exemplo, dos sistemas de atenção psicossocial a dependentes químicos e, consequentemente dos familiares, né. Eu acho que é porque é um tratamento conjunto, não é só da pessoa que é dependente químico, que está fazendo uso excessivo, é de todo o contexto daquela pessoa* ^(PP13)^.

*Causar mais acidentes, tanto de trânsito, de pessoas dirigirem alcoolizadas. Acidentes relacionados à violência doméstica, mortes e, até mesmo na rua, por brigas* ^(PB23)^.

As mudanças de comportamento podem afetar negativamente as relações familiares, de amizade e no ambiente de trabalho, devido à agressividade, impulsividade e ações inadequadas. A ausência emocional e física contribui para o afastamento de pessoas próximas. A deterioração da imagem social e profissional começam a se tornar evidentes, com prejuízo das atividades diárias e dificuldade em cumprir as responsabilidades.

*Quando as pessoas estão assim, elas passam a maior parte do tempo alteradas, elas também perdem muitas coisas. Perdem momentos em família, perdem essas coisas que aí que estaria ligado ao bem-estar emocional* ^(PE10)^.

*Perde a noção de pudor, né? Sei lá. E perde a noção de respeito do espaço dos outros. Acho que gera incômodo* ^(PP11)^.

O consumo contínuo pode comprometer a situação econômica do usuário excessivo, uma vez que, provavelmente irá utilizar boa parte da sua renda para comprar bebidas alcoólicas, comprometendo outras necessidades básicas. Pode interferir diretamente na capacidade para alcançar objetivos pessoais e profissionais, além de reduzir a produtividade e o desempenho nas atividades cotidianas.

*Gastar o dinheiro que tem em bebida, afeta tanto, né? Vai acabar afetando a saúde das pessoas. Ela vai gastar dinheiro na bebida e depois não vai ter o que comer* ^(PB23)^.

*A pessoa pode não conseguir focar no que ela tem que fazer, porque ela fica muito dependente do álcool. Ela está constantemente com vontade de tomar bebida alcoólica, então ela não consegue se concentrar em fazer outra coisa, como estudar ou fazer exercício físico* ^(PM24)^.

Os participantes PB1, PB3, PP4, PP6, PE8, PP12, P1P3, PE15, PP16, PP19, PM21, PB23, PM24, PB25, PE26, PE27 e PB28 referiram que o consumo excessivo de álcool pode prejudicar a saúde da pessoa ao longo da vida, devido aos riscos que fica exposta, como problemas hepáticos, cardíacos, neurológicos, renais, maior predisposição a diversos tipos de câncer, coma alcoólico, morte prematura, dentre outros. Outro aspecto significativo é a incapacidade para construir projetos de vida para o futuro e alcançar metas positivas de vida. Tais fatores limitam o bem-estar e uma vida saudável.

*Não afeta somente no momento que a pessoa está bebendo, né? Por exemplo, a cirrose, a pessoa para de beber por algum tempo, ela ainda vai sentir no futuro os efeitos disso, né? Ou então ela pode acabar tendo pré-disposição para outras doenças por causa desse período em que ela bebeu demais* ^(PP4)^.

*O consumo excessivo de álcool pode estressar as pessoas. Então pode levar a ter relações mais frágeis, conflituosas e distantes, assim como ocorrer rompimentos a longo prazo* ^(PP6)^.

Na maioria das vezes, o processo de aprendizagem fica prejudicado, uma vez que a pessoa que faz uso excessivo de bebidas alcoólicas não consegue realizar as atividades propostas, podendo chegar na situação extrema de perder totalmente sua funcionalidade pessoal e profissional.

*As perspectivas de uma vida futura ficam prejudicadas, eu acho que a pessoa perde um pouco a noção de onde ela quer chegar, talvez ela fique fechada ali naquele mundo de só fazer festa, beber e tudo mais, e aí eu acho que ela perde um pouco a perspectiva de onde quer chegar, ou, às vezes, até são pessoas que já não sabem mais onde querem chegar e, por isso, acabam entrando no mundo da bebida e a bebida se torna um conforto para os sofrimentos* ^(PE10)^.

Em relação à saúde mental, os participantes PB1, PP6, PP7, PE8, PP9, PP14, PE15, PB18, PM21, PM22, PM24, PB25, PB28, PB29 e PB30 relataram que o uso excessivo de bebidas alcoólicas pode afetar as estruturas cerebrais da pessoa, o que talvez possa criar a pré-disposição para o desencadeamento de transtornos mentais, como depressão, ansiedade, estresse intenso e dependência química.

*Qualquer problema, qualquer coisa, tu vais tentar buscar essa felicidade momentânea e cada vez tu vais aumentar mais a dose para tentar suprir o que está faltando na tua vida. Como qualquer vício. Vai começar baixo e tu vai acabar tendo que ingerir mais quantidade para chegar onde tu queres* ^(PB28)^.

O consumo excessivo de bebidas alcoólicas também é considerado pelos participantes como responsável pela redução significativa da qualidade de vida futura da pessoa, principalmente no período do envelhecimento.

*Eu acho que com certeza beber muito vai diminuir a expectativa de vida da pessoa, ela vai ser um idoso com mais problemas de saúde, com menor capacidade de locomoção, de independência* ^(PE15)^.

*Quando a pessoa estiver mais velha, ela vai perceber os efeitos do estilo de vida dela, mais lá na frente. Ela pode sentir que sua qualidade de vida reduziu muito no período do envelhecimento* ^(PM21)^.

## DISCUSSÃO

Apesar da diversidade dos participantes, os resultados do estudo evidenciaram que todos apresentavam dificuldades quanto à compreensão do conceito de consumo excessivo de álcool e o padrão de consumo considerando como excessivo. De acordo com o *National Institute on Alcohol Abuse and Alcoholism* (NIAAA), o consumo excessivo é caracterizado por um padrão de ingestão alcoólica que ultrapassa os limites considerados moderados, elevando o risco de problemas de saúde e outras consequências negativas. Essa definição compreende categorias específicas, como o *binge drinking* ou consumo episódico excessivo, definido como o consumo de cinco ou mais doses de bebidas alcoólicas para homens, ou quatro ou mais para mulheres, em um curto período de tempo (cerca de duas horas) e o *heavy drinking* ou consumo pesado, que se refere à ingestão semanal de 15 ou mais doses para homens, e 8 ou mais para mulheres^(18)^. Em relação à dose padrão, no Brasil, ainda não há um padrão oficial estabelecido, mas o Centro de Informação sobre Saúde e Álcool (CISA) utiliza a definição do NIAAA, que considera uma dose padrão como contendo 14 gramas de álcool puro, ou seja, o equivalente a 355 ml de cerveja (5% de teor alcoólico), 150 ml de vinho (12%) ou 45 ml de destilado, como vodca, uísque, cachaça, gin ou tequila (40%)^(11)^.

Contudo, os participantes do estudo demonstraram interpretar o consumo excessivo principalmente com base na frequência e não na quantidade consumida em determinado momento da vida cotidiana. Tal concepção revela uma lacuna na compreensão dos riscos específicos associados à ingestão rápida e elevada de álcool, mesmo que essa prática não seja diária. Além disso, embora o estudo tenha sido realizado sem o uso de uma escala de medição específica, as respostas dos participantes sugerem que eles não conhecem a quantidade exata correspondente a uma dose de bebidas alcoólicas que pode ser prejudicial à saúde. A falta de conhecimento sobre a quantidade exata de bebida pode ter várias consequências para o consumo de adultos universitários emergentes. Sem pontos de referência, eles podem consumir quantidades excessivas sem ter consciência disso, o que aumenta o risco de intoxicação por álcool e de comportamentos de risco. Em um ambiente universitário, onde o consumo de bebidas alcoólicas é frequentemente influenciado pelo contexto social, a falta de informações pode levar à adoção de normas sociais como base para a avaliação das quantidades ingeridas, em vez de uma avaliação pautada nos padrões de consumo.

A Teoria Bioecológica do Desenvolvimento Humano, particularmente no que se refere à pessoa, abrange três dimensões interdependentes, ou seja, as disposições, os recursos e as características biológicas do indivíduo, o que possibilita compreender melhor os resultados deste estudo. As disposições referem-se a tendências internas da pessoa, tais como: motivação, curiosidade e as escolhas comportamentais, principalmente no que se refere ao consumo de álcool. Neste contexto, observa-se uma tendência à normalização do consumo excessivo de bebidas alcoólicas em curtos períodos de tempo, especialmente em contextos sociais, onde o consumo elevado de álcool é, na maioria das vezes, incentivado e socialmente valorizado. A falta de conhecimento relativa aos prejuízos do consumo de álcool para a vida, altera a capacidade de autorregulação, a qual fica diminuída. Isso também aumenta a vulnerabilidade da pessoa a pressões externas. Tais disposições favorecem uma tolerância psicológica que dificulta a receptividade às mensagens de prevenção e limita o questionamento das condutas individuais.

Quanto aos recursos, Bronfenbrenner define-os como o conhecimento, as capacidades cognitivas e as competências que orientam as pessoas nas suas escolhas em seu ambiente. Assim, a ausência de uma percepção clara dos limites de risco e a compreensão fragmentada dos efeitos do consumo de álcool indicam a carência de informações específicas, fundamentais para a autorregulação e a prevenção eficaz entre adultos emergentes universitários. Essa limitação contribui para uma avaliação distorcida do próprio comportamento, gerando um conhecimento limitado sobre os mecanismos que ligam o consumo excessivo a consequências biopsicossociais graves, incluindo infecções transmissíveis e resistência a tratamentos médicos.

As características biológicas, por sua vez, referem-se a fatores fisiológicos, genéticos e do desenvolvimento que influenciam a resposta individual ao álcool. A literatura científica diferencia os limites de consumo conforme o sexo pois, as mulheres são mais vulneráveis aos efeitos do álcool devido as diferenças metabólicas e corporais^(19,20)^. Apesar disso, nenhuma das 23 mulheres participantes do estudo mencionou essa vulnerabilidade em suas respostas, o que provavelmente evidencia a ausência de compreensão sobre os riscos específicos relacionados ao gênero. Considerando que os adultos emergentes universitários estão na transição da adolescência para a vida adulta, fase em que o cérebro ainda está em desenvolvimento, o consumo excessivo pode comprometer processos neurológicos e cognitivos^(21,22)^. Essa variável tampouco foi considerada pelas participantes, o que reforça a falta de conhecimento relativas as influências das características biológicas e seu comportamento de consumo.

Em relação aos riscos para a saúde e o bem-estar biopsicossocial, os participantes apontaram algumas consequências a curto prazo, como fadiga, arrependimentos e alterações nas interações sociais. No entanto, os efeitos a longo prazo, apesar de mencionados, foram limitados a doenças hepáticas e renais, acidentes automobilísticos e conflitos familiares. A análise sugere uma compreensão parcial dos impactos do consumo excessivo de álcool, com escassez de informações e ausência de conexões entre causas, mecanismos e efeitos do uso. Esse conhecimento fragmentado não contempla de maneira clara os danos sociais, econômicos e de saúde pública implicados no uso abusivo de álcool, nem os entraves que representa para alcançar os Objetivos de Desenvolvimento Sustentável (ODS).

A análise das disposições revela que a motivação, as curiosidades e escolhas em relação ao consumo estão diretamente relacionadas aos contextos sociais, inclusive o familiar. A atenção centrada apenas na frequência de consumo ignora o risco representado pela ingestão elevada em um curto período de tempo, com pouca ou nenhuma percepção sobre os efeitos imediatos e acumulativos. Porém, por serem estudantes universitários, os participantes parecem mais propensos a práticas de consumo excessivo^(8)^, reforçando a necessidade de intervenções educativas específicas. Diante das adversidades cotidianas relativas às questões acadêmicas, sentimentos como tristeza, ansiedade e depressão são comuns, e, na ausência de recursos internos e externos para manterem a resiliência, o álcool pode ser usado como uma estratégia de relaxamento, tentando diminuir o desconforto emocional^(7)^. Essa realidade foi identificada nos relatos dos participantes, mas sem o aprofundamento necessário para reconhecer os mecanismos de vulnerabilidade e suas implicações a longo prazo.

Por fim, considerando que a maioria dos adultos emergentes universitários desta pesquisa irão atuar na área da saúde e da educação, torna-se essencial que adquiram uma compreensão científica e aprofundada relativa aos efeitos deletérios do consumo abusivo de álcool. Essa compreensão deve ir além do conhecimento empírico e, por isso deve incluir os aspectos biopsicossociais, fatores de risco individuais e contextuais, e implicações para a saúde coletiva, a fim de que possam desempenhar um papel ativo na promoção da sua saúde e das comunidades com as quais interagem.

Alguns pesquisadores analisaram o conhecimento dos riscos e das unidades padrão de álcool em quantidades específicas de cerveja, vinho e destilados. Atitudes e práticas realizadas pelos adultos emergentes universitários que usam tais bebidas ocorrem, com desconhecimento do padrão de consumo considerado excessivo. Os resultados encontrados comprovaram que o consumo de álcool é uma prática comum entre os adultos emergentes universitários dos cursos de graduação, mas os mesmos, incluindo os da área da saúde mostraram ter pouco conhecimento sobre seus prejuízos para a vida. Um estudo realizado na África do Sul com estudantes de graduação, cujo objetivo foi avaliar o conhecimento, as atitudes e as práticas relacionadas ao consumo de bebidas alcoólicas, revelou que aproximadamente um terço dos participantes não respondeu ao questionário referente às unidades padrão de consumo de álcool em diferentes tipos de bebidas, como cerveja, vinho e destilados. Entre os que responderam, 71,3% subestimaram o valor correspondente à unidade padrão^(23)^. De modo semelhante, um estudo transversal conduzido na Polônia com 480 universitários, com idades entre 18 e 35 anos, identificou um nível reduzido de conhecimento sobre os efeitos do álcool para a saúde^(24)^.

Estudo realizado em Portugal com adultos universitários emergentes, evidenciou que, em geral, eles tinham baixa pontuação relativa ao conhecimento sobre as consequências negativas do álcool para a saúde e o bem-estar. Entretanto, aqueles com maior pontuação de conhecimento consumiam mais álcool do que aqueles com menor pontuação^(25)^. Isso pode ocorrer devido à falta de conhecimento sobre os limites de consumo que podem afetar a saúde. Na Itália, em um estudo com 1928 estudantes universitários entre 18 a 24 anos, os resultados mostraram que os participantes do estudo não estavam totalmente cientes das consequências negativas do consumo excessivo de bebidas alcoólicas^(26)^. E, estudo realizado na China apresentou resultados semelhantes^(27)^.

A maioria das pesquisas relacionadas ao consumo de álcool são quantitativas e abordam os usuários em geral. Os aspectos diferenciados da maioria dos estudos realizados sobre este tema, na presente pesquisa estão relacionados a abordagem qualitativa, a qual permitiu que os adultos emergentes universitários pudessem revisitar um pouco da sua história de vida e refletir acerca da construção de conceitos e paradigmas relativos ao consumo de álcool, assim como repensar sobre a repercussão do uso para sua trajetória acadêmica e profissional. Outro diferencial foi a população específica pesquisada, ou seja, os adultos emergentes universitários. Os resultados evidenciaram a relevância deste tipo de pesquisa, pois permitiu realizar um aprofundamento do conhecimento e conscientização quanto a percepção dos riscos e o padrão de consumo excessivo, o impacto para sua saúde biopsicossocial, seu bem-estar e, assim, repensar seu uso, uma vez que emergiram questionamentos relativos ao seu comportamento e o quanto ele já estava prejudicando seu rendimento acadêmico, assim como, caso continuassem o padrão atual, com certeza teriam afetadas as futuras oportunidades profissionais.

## CONCLUSÃO

Os resultados mostraram que os participantes não sabem com profundidade quais as repercussões negativas e os riscos do uso excessivo de bebidas alcoólicas para a saúde biopsicossocial e bem-estar das pessoas; a quantidade de copos considerada padrão para definir o uso excessivo, o fato que o cérebro ainda se encontra em desenvolvimento e a relação entre gênero e álcool.

Como contribuição para a área da saúde, os resultados poderão orientar o planejamento de ações que incluam a conscientização e educação em saúde sobre os prejuízos do consumo excessivo do álcool para a saúde e qualidade de vida, abordando questões como: a definição de consumo excessivo; o que constitui uma dose padrão e a quantidade de álcool (em mililitros) correspondente a uma dose, dependendo do tipo de bebida; diferenças de gênero; consequências socioeconômicas e para a saúde; sinais de dependência do álcool; os motivos fisiológicos pelos quais o álcool é proibido para menores de idade, dentre outros aspectos relevantes, especificamente para adultos emergentes universitários.

Além da necessidade de realizar campanhas de conscientização e educação em saúde, as quais são fundamentais para que os adultos emergentes universitários aprendam como administrar seu tempo e o estresse de forma saudável a fim de manter sua saúde mental. Entende-se como importante que os profissionais de saúde, ao realizarem tais campanhas, enfatizem às razões e os meios de controlar o consumo de álcool. Mostrar exemplos reais das repercussões negativas do uso de bebidas alcoólicas para a saúde biopsicossocial e para a qualidade de vida a médio e longo prazo, provavelmente irá incentivar a compreensão sobre as advertências e restrições do uso, mostrando que essas restrições não têm a intenção de diminuir a liberdade ou prazer, mas evitar as consequências negativas para a vida e para as pessoas ao seu redor.

Como limitações do presente estudo incluem-se o fato da maioria dos estudantes universitários serem mulheres, as quais, geralmente tem uma visão diferenciada dos homens em relação ao consumo. Além disso, os dados foram coletados em um contexto sociocultural específico, o que pode influenciar as percepções dos participantes sobre o consumo excessivo de álcool e, o mesmo foi realizado em apenas uma instituição de ensino superior, com um número pequeno de participantes, por ser uma pesquisa qualitativa e, desta forma priorizou as vivências, experiências e subjetividades dos estudantes.

Enfim, considera-se que as lacunas encontradas abrem novas possibilidades para pesquisas futuras sobre o consumo de álcool por adultos emergentes universitários e podem gerar reflexões sobre o papel das políticas relativas ao uso de álcool e outras drogas e, também à função social das universidades, a fim de proporcionar maior qualidade de vida para esta população específica que, em sua maioria, passa a formar parte da força produtiva de trabalho do país.

## Data Availability

Os dados que fundamentam este estudo foram depositados no repositório SciELO Data, disponível para acesso por meio do seguinte endereço eletrônico: https://data.scielo.org/dataset.xhtml?persistentId=doi:10.48331/SCIELODATA.RU7N8N.

# Translation: Adultos emergentes universitarios y el conocimiento relativo a las consecuencias del consumo excesivo de bebidas alcohólicas

**DOI:** 10.1590/1980-220X-REEUSP-2025-0088es

**Authors:** Roselande Simon, Adriane Maria Netto de Oliveira, Cristiane Barros Marcos, Mara Regina Santos da Silva, Dominique Therrien, Patrícia Bitencourt Toscani Greco, Evy Nazon, Mara Regina Pombo Amaral, Émilen Vieira Simões

**Affiliations:** 1 Universidade Federal do Rio Grande, Programa de Pós-Graduação em Enfermagem, Rio Grande, RS, Brasil.; 2 Universidade Federal do Rio Grande, Instituto de Ciências Biológicas, Rio Grande, RS, Brasil.; 3 Université du Québec en Outaouais, Département des Sciences Infirmières, Gatineau, Québec, Canadá.

**Keywords:** Consumo de Bebidas Alcohólicas, Estudiantes, Salud Humana, Bienestar Psicológico

## Abstract

**Objetivo:**

Comprender la percepción y el conocimiento de los adultos universitarios emergentes sobre las consecuencias del consumo excesivo de bebidas alcohólicas para la salud biopsicosocial y el bienestar actual y futuro de las personas.

**Método:**

Se trata de un estudio exploratorio, descriptivo y de enfoque cualitativo, realizado con 31 estudiantes universitarios. Se difundió en línea un formulario con código QR para informar sobre la investigación. La recolección de datos se llevó a cabo mediante entrevistas semiestructuradas. Los datos se transcribieron con el software Turbo Scribe y los discursos se analizaron con la ayuda del software Atlas.ti, utilizando la técnica de análisis de contenido.

**Resultados:**

Existe una laguna en el conocimiento de los participantes sobre qué es el consumo excesivo de bebidas alcohólicas y sus repercusiones para la salud. Se concluye que es fundamental promover campañas continuas de concienciación y educación en salud, con el fin de ampliar el conocimiento y favorecer decisiones conscientes sobre el consumo de alcohol.

## INTRODUCCIÓN

El alcohol es la sustancia psicoactiva más consumida en el mundo a través de bebidas alcohólicas. El consumo puede ser moderado, excesivo o nocivo y provocar dependencia alcohólica. Según la Organización Mundial de la Salud (OMS), todo consumo de alcohol es perjudicial para la salud y la vida de las personas^(1)^. Los estudios demuestran que el consumo de bebidas alcohólicas contribuye al aumento global de la prevalencia de enfermedades no transmisibles, accidentes de tráfico, violencia doméstica, violencia física, violencia psicológica, abuso sexual y aumento de la mortalidad. Esto eleva los gastos en asistencia sanitaria, los costes sociales y legales^(2,3)^. Por estas razones, se considera un grave problema de salud pública y una de las causas de muerte evitable.

En todos los grupos de edad, los adultos emergentes son los que tienen una tasa de consumo más alta y una tasa de abstinencia más baja^(4,5)^. El adulto emergente es un concepto definido por el psicólogo estadounidense Jeffrey Jensen Arnet en 2000 como las características específicas del desarrollo de los jóvenes de entre 18 y 25 años. Las cinco características específicas son: la exploración de la identidad, periodo durante el cual el joven busca la respuesta a la pregunta «quién soy yo», ya sea en términos personales, relacionales y/o profesionales. La segunda se refiere a la inestabilidad, que se explica por el hecho de que, durante esta fase, los jóvenes aún no aún no han alcanzado la estabilidad propia de la vida adulta. Con frecuencia, están explorando opciones profesionales, cambian de residencia, y sus metas y relaciones tienden a transformarse varias veces hasta que logran definir con mayor claridad lo que desean para sí mismos. La tercera característica se define como la edad que exige aún más responsabilidades, lo que a menudo genera una sobrecarga en la vida, ya que este es el período en el que se establecen las bases para la calidad de vida en la etapa adulta. La cuarta característica está relacionada con el sentimiento de ambivalencia, que muestra que estos jóvenes ya no son adolescentes, pero tampoco son aún adultos. Y la quinta característica se refiere a una edad de muchas posibilidades, por lo que este período está marcado por grandes oportunidades de preparación para una buena calidad de vida durante el ciclo vital^(6)^.

En la población de adultos emergentes, la literatura muestra que aquellos que asisten a la universidad tienen una mayor prevalencia de consumo de bebidas alcohólicas en comparación con los jóvenes de la misma edad que no son estudiantes universitarios. Probablemente esto se deba a que participan en actividades o eventos que facilitan el consumo excesivo de alcohol, como fiestas, juegos y actividades de ocio entre estudiantes universitarios^(7,8)^. La adquisición de conocimientos sobre las consecuencias negativas del consumo excesivo de alcohol y sobre lo que se considera un consumo moderado puede permitir un uso moderado de esta sustancia psicoactiva, estimulando a los adultos emergentes universitarios a adoptar hábitos saludables en su vida cotidiana. Otro aspecto se refiere a los factores de riesgo que, al ser reconocidos, permiten tomar decisiones de forma consciente^(9)^.

La información relacionada con las consecuencias negativas del consumo excesivo de bebidas alcohólicas, así como el patrón que lo define, puede ayudar a los adultos emergentes a determinar si este comportamiento vale la pena, incluso en comparación con los riesgos que conlleva. Probablemente, el conocimiento facilita que las personas prioricen acciones más saludables en su vida cotidiana, con el fin de mantener la salud biopsicosocial y el bienestar, tanto propio como el de su familia y comunidad^(10)^.

Los estudios muestran que el grupo de pares ejercen una gran influencia en el consumo de bebidas alcohólicas por parte de los adultos emergentes universitarios^(7,8)^. Así, cuando se relacionan con ellos, hacen un uso moderado y tienden a adoptar y mantener el mismo comportamiento.

Según el último informe de la OMS, el consumo de bebidas alcohólicas en 2019 fue responsable de alrededor de 2,6 millones de muertes. Además, el 7% de la población mundial de 15 años o más tiene al menos un trastorno mental relacionado con el consumo de alcohol, siendo el más prevalente la dependencia química, con una tasa del 3,7%^(1)^. En Brasil, se registraron 50 000 casos de hospitalización en el año 2023, y la tasa de muertes atribuibles al alcohol por cada 100 000 habitantes fue del 33% en 2022^(11)^.

En Brasil, los costes sociales del consumo de alcohol ascendieron a 18 800 millones de reales en 2019^(12)^. A la vista de estos datos, es probable que promover el conocimiento sobre las consecuencias negativas del consumo excesivo y la cantidad de unidades que lo definen sea una inversión relevante y sostenible. A partir de esta información y concienciación, diversas autoridades sanitarias, educativas, políticas, sociales, de seguridad pública, entre otras, podrán orientar acciones para la implementación de campañas que construyan bases sólidas para fomentar el consumo moderado. Estas acciones pueden contribuir positivamente a la salud biopsicosocial y al bienestar general de las personas, incluidos los adultos universitarios emergentes.

Según las simulaciones de los países de la Organización para la Cooperación y el Desarrollo Económico (OCDE), los costes socioeconómicos del consumo de alcohol entre 2020 y 2050 podrían alcanzar los 138 000 millones de dólares estadounidenses al año, a lo que se suma una pérdida de aproximadamente 33 millones de trabajadores y una caída del 1,6% del PIB^(13)^. Estos datos justifican la realización del presente estudio. Por otra parte, aunque el consumo de bebidas alcohólicas entre los jóvenes universitarios ya ha sido ampliamente estudiado, pocos trabajos se han dedicado a examinar el conocimiento de los adultos emergentes universitarios sobre lo que constituye un consumo excesivo, así como sobre las implicaciones de dicho consumo para la salud y el bienestar, tanto en el presente como en el futuro. Por lo tanto, el objetivo de este artículo es comprender la percepción y el conocimiento de los adultos emergentes universitarios sobre las consecuencias del consumo excesivo de bebidas alcohólicas para la salud biopsicosocial y el bienestar actual y futuro de las personas.

## MÉTODO

### Tipo de Estudio

Este estudio es un extracto de la tesis de maestría titulada: «Transición a la edad adulta emergente y consumo de bebidas alcohólicas: desde una perspectiva bioecológica». Se trata de un estudio con un enfoque cualitativo, exploratorio y descriptivo, guiado por las directrices de los Criterios Consolidados para la Presentación de Informes de Investigación Cualitativa (Consolidated Criteria for Reporting Qualitative Research- COREQ), que cumple con los 32 puntos establecidos en el checklist^(14)^. La elección de este enfoque se justifica por su objetivo, ya que este tipo de investigación se centra en la comprensión y descripción de problemas y fenómenos complejos y dinámicos, contribuyendo a una mejor comprensión del objeto de estudio. Además, permite a los investigadores comprender la diversidad y la riqueza de las experiencias humanas^(15)^.

### Contextos del Estudio

El estudio se llevó a cabo en la Universidad Federal de Rio Grande (FURG), situada en el municipio de Rio Grande/RS, en el extremo sur de Brasil. Los contextos de investigación fueron la Unidad de Salud del Área Académica y, en concreto, la Facultad de Medicina (FAMED) y el Curso de Graduación en Medicina; el Campus Carreiros, el Instituto de Ciencias Biológicas (ICB), concretamente el Grado en Biología; la Escuela de Ingeniería (EE-FURG), concretamente el Grado en Ingeniería Civil, y, por último, el Instituto de Ciencias Humanas e de la Información (ICHI), concretamente el Grado en Psicología.

### Participantes y Criterios de Selección

Los participantes fueron seleccionados mediante un método de muestreo no probabilístico. En total, participaron 31 estudiantes universitarios: 9 del Grado en Biología; 6 del Grado en Ingeniería Civil; 12 del Grado en Psicología y 4 del Grado en Medicina. En cuanto al género, 23 participantes se identificaron como mujeres y 8 como hombres. Con este número se alcanzó la saturación de los datos, ya que las respuestas obtenidas se volvieron repetitivas y todas las preguntas de la investigación ya habían sido contempladas. Se adoptaron como criterios de inclusión: que el estudiante tuviera entre 18 y 24 años; estuviera matriculado regularmente en el Curso de Licenciatura en Biología, Medicina, Psicología e Ingeniería Civil y que consumiera o hubiera consumido bebidas alcohólicas en los últimos doce meses hasta el momento de la Recolección de datos. Este grupo de edad forma parte de un proceso de adaptación al contexto sociocultural de Brasil, que determina que, entre las edades mencionadas en este periodo de la vida, el grupo de entre 18 y 24 años, comúnmente conocido como jóvenes-jóvenes, se encuentra dentro del referente utilizado sobre los adultos emergentes^(16)^. Los criterios de exclusión fueron: los estudiantes que no pudieron participar en la entrevista debido a la realización de exámenes, pruebas o en función de la realización de otras actividades académicas y aquellos que no se presentaron a la entrevista en tres ocasiones consecutivas.

### Recolección de Datos

Los datos se recolectaron del 17 de septiembre al 24 de octubre de 2024, mediante una entrevista semiestructurada. Para contactar a los estudiantes universitarios, se preparó un formulario con un código QR, en el que se explicaban los objetivos de la investigación y se solicitaba el lugar y la mejor hora para realizar la entrevista con los participantes. Los formularios se compartieron a través de las redes sociales, principalmente Instagram y WhatsApp. Aquellos que quisieron participar escanearon el código QR para acceder a la información de la investigación. Otra forma de solicitar la participación de los estudiantes universitarios fue de forma presencial, cuando la estudiante de máster fue a las aulas para hablar sobre el estudio y animarlos a participar. En total, 57 estudiantes completaron el formulario. De ellos, se seleccionaron los dos primeros participantes para validar el guion de la entrevista semiestructurada. Nueve desistieron y, además, se excluyeron 24 de la muestra: 10 por tener más de 24 años, 5 por no haber consumido alcohol en los últimos 12 meses, 2 desistieron tras leer el TCLE y 7 por no tener tiempo para participar en la entrevista. Las entrevistas se realizaron con 31 participantes, en un lugar reservado, garantizando la privacidad y el anonimato de los participantes.

Antes de iniciar las entrevistas, la estudiante de maestría (primera autora) del Programa de Posgrado en Enfermería (PPGEnf) se presentó y explicó los objetivos y la metodología de la investigación. A continuación, a aquellos que aceptaron participar se les entregó el Término de Consentimiento Libre y Esclarecido (TCLE) en dos ejemplares para que lo firmaran, quedando una copia con ellos y otra con la estudiante de maestría. Las entrevistas duraron entre 26 y 70 minutos y se grabaron utilizando la aplicación de *Play Store*. Los participantes fueron identificados con la letra «P», que se numeró a medida que se realizaban las entrevistas, es decir, el primer participante se registró como número P1, el segundo como P2, y así sucesivamente hasta el participante 31. A continuación, se añadió la primera letra del curso, siendo B para Biología, E para Ingeniería Civil, P para Psicología y M para Medicina.

### Análisis y Tratamiento de Los Datos

Los datos se analizaron mediante el análisis de contenido temático de Minayo. Se trata de una combinación de procedimientos de análisis de la comunicación, con la idea de describir el contenido de los mensajes mediante etapas sistemáticas, para obtener la información necesaria para la realización de la investigación. Este tipo de análisis considera un tema específico del conocimiento.

El análisis temático se compone de tres fases: (a) preanálisis, cuando se realiza la lectura del material recolectado; (b) exploración del material, período en el que se inicia el análisis de los datos; (c) tratamiento e interpretación de los resultados, cuando se lleva a cabo la discusión de los datos^(15)^. En la etapa inicial, las entrevistas fueron transcritas con la ayuda del software Turbo Scribe, revisadas con base en los audios y organizadas para formar el corpus final. A partir de la lectura comprensiva de los discursos, se elaboraron categorías iniciales basadas en los constructos de la teoría bioecológica del desarrollo humano de Bronfenbrenner. Esta teoría propone una comprensión del desarrollo humano basada en las interacciones dinámicas entre el individuo y varios sistemas ambientales a lo largo del tiempo. Tiene cuatro componentes centrales: Proceso, Persona, Contexto y Tiempo. En este trabajo, el énfasis se pone en el componente persona, que se refiere a las características individuales (disposiciones, recursos y demandas) que cada sujeto aporta a sus experiencias en el mundo y que influyen en la forma en que percibe, responde y se adapta al entorno^(17)^. La adopción de esta perspectiva se basa en la hipótesis de que estas características influyen directamente en la percepción y la comprensión de los jóvenes sobre lo que es el consumo excesivo de alcohol, la dosis considerada excesiva y las consecuencias para la salud. Este enfoque permitió destacar la diversidad de representaciones y conocimientos de los participantes, teniendo en cuenta sus características individuales. Las categorías construidas a partir del análisis de los datos están directamente relacionadas con este componente.

En la segunda fase, que corresponde a la exploración del material, los discursos de los participantes se agruparon por categorías, utilizando el software Atlas.ti. Inicialmente, los datos se categorizaron con los siguientes códigos: percepción relativa al consumo; conocimiento sobre el consumo excesivo y el patrón de consumo que puede afectar a la salud biopsicosocial; conocimiento relativo a los impactos negativos del consumo excesivo. Posteriormente, las categorías se reagruparon de la siguiente manera: Percepción de los adultos emergentes sobre el consumo excesivo de bebidas alcohólicas; Conocimiento de los adultos emergentes sobre las consecuencias del consumo excesivo para la salud biopsicosocial y el bienestar.

En la tercera y última fase se llevó a cabo la discusión de los datos, a partir de conceptos y autores que trabajan con el tema propuesto en este estudio, así como utilizando los constructos de la teoría utilizada. Se envió a los participantes una invitación como feedback para informarles de los resultados del estudio realizado. Se utilizó Zotero como gestor de referencias.

### Aspectos Éticos

En relación con los aspectos éticos de la investigación, el estudio fue aprobado por el Comité de Ética en Investigación de la Universidad Federal de Rio Grande (CEP/FURG) con el dictamen n.º 7.033.705 y CAAE: 81929224.5.0000.5324, siguiendo todas las recomendaciones de la Resolución CNS nº 510/2016 para investigaciones con seres humanos. La realización del estudio también fue autorizada por los coordinadores de los cursos de grado mencionados anteriormente.

## RESULTADOS

### Caracterización de los Participantes

En el estudio participaron treinta y un estudiantes con edades comprendidas entre los 18 y los 24 años. De ellos, el 29,03% tiene veintiún años, el 22,58% tiene veinte años, el 12,9% tiene veintidós años, el 12,9% tiene veintitrés años, el 12,9% tiene diecinueve años, el 6,45% tiene veinticuatro años y el 3,23% tiene dieciocho años. En cuanto al género, 23 se identificaron como mujeres y ocho como hombres. Todos se declararon solteros. Nueve viven con familiares (madre y/o padre), cuatro viven con su novia/novio y dieciséis viven solos. Doce son de la ciudad de Rio Grande, mientras que diecinueve pertenecen a otras localidades, según las ciudades y estados que se indican a continuación: Vitória da Conquista (BA); Brasília (DF); Goiás Velho (GO); Alegrete (RS), Bagé (RS), Camaquã (RS), Canguçu (RS), Estrela (RS), Gravataí (RS), Pedro Osório (RS), Pelotas (RS), Santa Maria (RS), Santana do Livramento (RS), São Gabriel (RS), Sarandi (RS), Tavares (RS) y Piracicaba (SP).

### Percepción de los Adultos Emergentes Sobre el Consumo Excesivo de Bebidas Alcohólicas

En esta categoría se destacó la percepción de los adultos emergentes universitarios sobre la noción de consumo excesivo y el patrón de lo que consideran excesivo, lo que permitió comprender si saben cuándo un consumo es excesivo y si conocen la cantidad que se considera perjudicial para la salud. Según los PB1, PB3, PP4, PP7, PE8, PE10, PP11, PP14, PP17, PM20, PM21, PB23, PB25, PE27 y PE31, el consumo excesivo es cuando la persona consume bebidas alcohólicas a diario o varias veces a la semana, tal y como se describe a continuación:

*Todos los días voy a una fiesta, todos los días me emborracho, para mí eso es un consumo excesivo. Todos los días bebo, porque me encanta la cerveza, eso es excesivo para mí. Hoy en día creo que la gente bebe mucha más cerveza que agua. Por lo tanto, entiendo el consumo excesivo como todos los días o alguna cantidad diaria, por mínima que sea^(PB25)^.*

*Creo que sería eso de todos los días y a cualquier hora. Por ejemplo, para mí es muy surrealista que alguien esté bebiendo alcohol a las dos de la tarde en pleno miércoles, ¿sabes? Entonces, desde el momento en que la persona empieza a beber por la mañana, por la tarde y por la noche, todos los días, el consumo descontrolado, para mí ya es excesivo^(PB3)^.*

En opinión de PP5, PP6, PP7, PP9, PM20, PB23 y PM24, el consumo excesivo está relacionado con el motivo por el que la persona consume bebidas alcohólicas, ya sea para aliviar el estrés, huir de la realidad de la vida o consumir sin autocontrol.

*Creo que debe ser una forma de escapar de la realidad. Para olvidar todos los problemas^(PB23)^.*

*Creo que es eso, consumir alcohol en momentos en los que no deberías, por ejemplo, cuando estás triste, para no deprimirte, o beber todos los días para relajarte^(PM24)^.*

En opinión de PE10, PP17, PP19 y PE27, el consumo excesivo es cuando la persona se siente mal después de consumir bebidas alcohólicas, es decir, presenta síntomas físicos desagradables como mareos, náuseas y vómitos, o cuando se vuelve incapaz de mantener el autocontrol y protegerse.

*Creo que en la medida en que empiezas a sentirte mareado, mal, incapaz de hacer cosas, creo que eso ya es perjudicial para la salud^(PP17)^.*

*Sigues bebiendo, bebiendo y empiezas a encontrarte mal, ¿sabes? Empiezas a vomitar. O, como si no supieras lo que está pasando. No sabes lo que estás haciendo. Ni siquiera puedes caminar derecho y sigues bebiendo^(PP19)^.*

En cuanto a la dosis estándar que puede afectar a la salud, los participantes PB1, PB3, PP4, PP6, PE8, PE10, PE15, PP16, PB18, PM20, PM22, PB25 y PE26 señalan que el consumo diario, independientemente de la cantidad ingerida, puede ser perjudicial para la salud, porque a lo largo de la vida, los efectos acumulativos del consumo desarrollan una dependencia química.

*Quizás una botella de vino, por ejemplo. O un par de botellas de cerveza a la semana. Creo que eso ya tendría algún impacto, en cierta medida^(PP4)^.*

*Es decir, beber un vaso de cerveza todos los días ya es mucho, ¿entiendes? Una vez que tienes esa rutina, un vaso al día ya no es nada, y entonces empieza a aumentar la cantidad de alcohol que se consume al día^(PE8)^.*

Los participantes PP16, PB23, PM24, PB25 y PE26 informaron que desconocen la cantidad de consumo de bebidas alcohólicas que puede ser perjudicial para la salud.

*La cantidad, no lo sé. Pero la frecuencia, creo que si bebes todas las semanas, dos veces por semana es mucho^(PM24)^.*

*Creo que la frecuencia diaria puede ser perjudicial para la salud. Y la cantidad, no lo sé. Sé lo de la frecuencia, porque ahí entra en juego el alcoholismo^(PE26)^.*

Solo los participantes PP5, PP9, PP14, PM20 y PM21 mencionaron explícitamente tanto la frecuencia como la cantidad como un consumo o patrón de uso que puede ser perjudicial para la salud.

*Creo que el consumo de alcohol, al menos en cantidades mayores, una vez a la semana. Una o dos veces a la semana en grandes cantidades, o todos los días, ya es perjudicial^(PP5)^.*

Creo que hay dos tipos de consumo, uno muy frecuente, aunque sea en poca cantidad, pero muy frecuente, y otro muy intenso, pero no frecuente. Claro, un consumo intenso y frecuente creo que es el peor escenario. Pero creo que incluso si es tres veces al mes, te emborrachas, te desmayas, no recuerdas nada, ya creo que no es un consumo saludable, pero también todos los días, si tomas dos o tres copas de alguna bebida, también creo que no es un consumo saludable a largo plazo^(PP9)^.

### Conocimiento de los Adultos Emergentes Sobre las Consecuencias del Consumo Excesivo Para la Salud Biopsicosocial y el Bienestar

En esta categoría se recoge la información o los conocimientos que los adultos emergentes universitarios tienen sobre los efectos que el consumo excesivo de alcohol tiene sobre la salud biopsicosocial y el bienestar actual y futuro del consumidor y de las personas de su entorno. Los discursos de los estudiantes universitarios muestran que el consumo excesivo puede comprometer las relaciones profesionales y sociales, así como afectar la salud física y mental de los consumidores con consecuencias negativas inmediatas y a lo largo de la vida, incluyendo la sobrecarga del sistema de salud y los servicios sociales debido a los problemas sociales y financieros.

Los participantes PB1, PB3, PP5, PP6, PP7, PP9, PE10, PP12, PP13, PP14, PP17, PB18, PM20, PM21, PM22, PB23, PM24 y PE27 informan que el consumo excesivo puede afectar a la sociedad, debido a los frecuentes accidentes de tráfico causados por conductores ebrios y enfermedades crónicas no transmisibles, con el consiguiente aumento de los costes de los servicios sanitarios y sociales.

*Sobrecarga del SUS (Sistema Único de Salud), por ejemplo, de los sistemas de atención psicosocial a los dependientes químicos y, en consecuencia, a sus familiares, ¿no? Creo que es porque se trata de un tratamiento conjunto, no solo de la persona que es adicta, que está haciendo un uso excesivo, sino de todo el contexto de esa persona^(PP13)^. Provocar más accidentes, tanto de tráfico, como de personas que conducen bajo los efectos del alcohol. Accidentes relacionados con la violencia doméstica, muertes e incluso en la calle, por peleas^(PB23)^.*

Los cambios de comportamiento pueden afectar negativamente a las relaciones familiares, de amistad y en el entorno laboral, debido a la agresividad, la impulsividad y las acciones inadecuadas. La ausencia emocional y física contribuye al alejamiento de las personas cercanas. El deterioro de la imagen social y profesional comienza a hacerse evidente, con perjuicio para las actividades diarias y dificultad para cumplir con las responsabilidades.

*Cuando las personas están así, pasan la mayor parte del tiempo alteradas, también pierden muchas cosas. Pierden momentos en familia, pierden esas cosas que estarían relacionadas con el bienestar emocional^(PE10)^.*

*Pierden el sentido del pudor, ¿no? No sé. Y pierden el sentido del respeto por el espacio de los demás. Creo que genera incomodidad ^(PP11)^.*

El consumo continuado puede comprometer la situación económica del consumidor excesivo, ya que probablemente destinará gran parte de sus ingresos a la compra de bebidas alcohólicas, comprometiendo otras necesidades básicas. Puede interferir directamente en la capacidad para alcanzar objetivos personales y profesionales, además de reducir la productividad y el rendimiento en las actividades cotidianas.

*Gastar el dinero que tienes en bebida afecta mucho, ¿no? Acaba afectando a la salud de las personas. Ella va a gastar dinero en bebida y luego no tendrá qué comer^(PB23)^.*

*La persona puede no ser capaz de concentrarse en lo que tiene que hacer, porque se vuelve muy dependiente del alcohol. Constantemente tiene ganas de beber alcohol, por lo que no puede concentrarse en hacer otras cosas, como estudiar o hacer ejercicio físico^(PM24)^.*

Los participantes PB1, PB3, PP4, PP6, PE8, PP12, P1P3, PE15, PP16, PP19, PM21, PB23, PM24, PB25, PE26, PE27 y PB28 mencionaron que el consumo excesivo de alcohol puede perjudicar la salud de las personas a lo largo de su vida, debido a los riesgos a los que se exponen, como problemas hepáticos, cardíacos, neurológicos, renales, mayor predisposición a diversos tipos de cáncer, coma alcohólico, muerte prematura, entre otros. Otro aspecto significativo es la incapacidad para construir proyectos de vida para el futuro y alcanzar metas positivas en la vida. Estos factores limitan el bienestar y una vida saludable.

*No solo afecta en el momento en que la persona está bebiendo, ¿verdad? Por ejemplo, en el caso de la cirrosis, aunque la persona deje de beber durante un tiempo, seguirá sintiendo los efectos en el futuro, ¿no? O bien, puede acabar teniendo predisposición a otras enfermedades debido al periodo en el que bebió en exceso^(PP4)^.*

*El consumo excesivo de alcohol puede estresar a las personas. Por lo tanto, puede llevar a relaciones más frágiles, conflictivas y distantes, así como a rupturas a largo plazo^(PP6)^.*

En la mayoría de los casos, el proceso de aprendizaje se ve perjudicado, ya que la persona que consume alcohol en exceso no puede realizar las actividades propuestas, pudiendo llegar a la situación extrema de perder totalmente su funcionalidad personal y profesional.

*Las perspectivas de una vida futura se ven perjudicadas, creo que la persona pierde un poco la noción de dónde quiere llegar, tal vez se encierra en ese mundo de solo fiesta, beber y todo eso, y ahí creo que pierde un poco la perspectiva de adónde quiere llegar, o, a veces, incluso son personas que ya no saben adónde quieren llegar y, por eso, terminan entrando en el mundo de la bebida y la bebida se convierte en un consuelo para sus sufrimientos^(PE10)^.*

En relación con la salud mental, los participantes PB1, PP6, PP7, PE8, PP9, PP14, PE15, PB18, PM21, PM22, PM24, PB25, PB28, PB29 y PB30 relataron que el consumo excesivo de bebidas alcohólicas puede afectar las estructuras cerebrales de la persona, lo que tal vez pueda crear una predisposición al desencadenamiento de trastornos mentales, como depresión, ansiedad, estrés intenso y dependencia química.

*Cualquier problema, cualquier cosa, vas a intentar buscar esa felicidad momentánea y cada vez vas a aumentar más la dosis para intentar suplir lo que te falta en la vida. Como cualquier adicción. Empezarás poco a poco y acabarás teniendo que ingerir más cantidad para llegar a donde quieres^(PB28)^.*

El consumo excesivo de bebidas alcohólicas también es considerado por los participantes como responsable de la reducción significativa de la calidad de vida futura de la persona, especialmente en el período de envejecimiento.

*Creo que, sin duda, beber mucho reducirá la esperanza de vida de la persona, que será un anciano con más problemas de salud, con menor capacidad de locomoción y de independencia^(PE15)^.*

*Cuando la persona sea mayor, se dará cuenta de los efectos de su estilo de vida, más adelante. Puede sentir que su calidad de vida se ha reducido mucho en el período de envejecimiento^(PM21)^.*

## DISCUSIÓN

A pesar de la diversidad de los participantes, los resultados del estudio pusieron de manifiesto que todos tenían dificultades para comprender el concepto de consumo excesivo de alcohol y el patrón de consumo considerado excesivo. Según el *Instituto Nacional sobre el Abuso del Alcohol y el Alcoholismo* (*National Institute on Alcohol Abuse and Alcoholism*-NIAAA), el consumo excesivo se caracteriza por un patrón de ingesta de alcohol que supera los límites considerados moderados, lo que aumenta el riesgo de problemas de salud y otras consecuencias negativas. Esta definición comprende categorías específicas, como el *binge drinking* o consumo episódico excesivo, definido como el consumo de cinco o más dosis de bebidas alcohólicas para los hombres, o cuatro o más para las mujeres, en un corto período de tiempo (alrededor de dos horas) y el *heavy drinking* o consumo pesado, que se refiere a la ingesta semanal de 15 o más dosis para los hombres y 8 o más para las mujeres^(18)^. En cuanto a la dosis estándar, en Brasil aún no existe una norma oficial establecida, pero el Centro de Información sobre Salud y Alcohol (CISA) utiliza la definición de la NIAAA, que considera que una dosis estándar contiene 14 gramos de alcohol puro, es decir el equivalente a 355 ml de cerveza (5% de alcohol), 150 ml de vino (12%) o 45 ml de destilados, como vodka, whisky, cachaça, ginebra o tequila (40%)^(11)^.

Sin embargo, los participantes en el estudio demostraron interpretar el consumo excesivo principalmente en función de la frecuencia y no de la cantidad consumida en un momento determinado de la vida cotidiana. Esta concepción revela una laguna en la comprensión de los riesgos específicos asociados al consumo rápido y elevado de alcohol, aunque esta práctica no sea diaria. Además, aunque el estudio se llevó a cabo sin utilizar una escala de medición específica, las respuestas de los participantes sugieren que no conocen la cantidad exacta de una dosis de bebidas alcohólicas que puede ser perjudicial para la salud. La falta de conocimiento sobre la cantidad exacta de bebida puede tener varias consecuencias para el consumo de los adultos universitarios emergentes. Sin puntos de referencia, pueden consumir cantidades excesivas sin ser conscientes de ello, lo que aumenta el riesgo de intoxicación por alcohol y de comportamientos de riesgo. En un entorno universitario, donde el consumo de bebidas alcohólicas suele estar influenciado por el contexto social, la falta de información puede llevar a la adopción de normas sociales como base para evaluar las cantidades ingeridas, en lugar de una evaluación basada en los patrones de consumo.

La Teoría Bioecológica del Desarrollo Humano, particularmente en lo que se refiere a la persona, abarca tres dimensiones interdependientes, es decir, las disposiciones, los recursos y las características biológicas del individuo, lo que permite comprender mejor los resultados de este estudio. Las disposiciones se refieren a las tendencias internas de la persona, tales como: motivación, curiosidad y elecciones conductuales, principalmente en lo que se refiere al consumo de alcohol. En este contexto, se observa una tendencia a la normalización del consumo excesivo de bebidas alcohólicas en períodos cortos de tiempo, especialmente en contextos sociales, donde el consumo elevado de alcohol es, en la mayoría de los casos, incentivado y valorado socialmente. La falta de conocimiento sobre los daños que el consumo de alcohol causa en la vida altera la capacidad de autorregulación, que se ve disminuida. Esto también aumenta la vulnerabilidad de la persona a las presiones externas. Estas disposiciones favorecen una tolerancia psicológica que dificulta la receptividad a los mensajes de prevención y limita el cuestionamiento de las conductas individuales.

En cuanto a los recursos, Bronfenbrenner los define como el conocimiento, las capacidades cognitivas y las competencias que orientan a las personas en sus elecciones en su entorno. Así, la ausencia de una percepción clara de los límites del riesgo y la comprensión fragmentada de los efectos del consumo de alcohol indican la falta de información específica, fundamental para la autorregulación y la prevención eficaz entre los adultos universitarios emergentes. Esta limitación contribuye a una evaluación distorsionada del propio comportamiento, generando un conocimiento limitado sobre los mecanismos que vinculan el consumo excesivo con consecuencias biopsicosociales graves, incluidas las infecciones transmisibles y la resistencia a los tratamientos médicos.

Las características biológicas, por su parte, se refieren a factores fisiológicos, genéticos y de desarrollo que influyen en la respuesta individual al alcohol. La literatura científica diferencia los límites de consumo según el sexo, ya que las mujeres son más vulnerables a los efectos del alcohol debido a las diferencias metabólicas y corporales^(19,20)^. A pesar de ello, ninguna de las 23 mujeres participantes en el estudio mencionó esta vulnerabilidad en sus respuestas, lo que probablemente pone de manifiesto la falta de comprensión de los riesgos específicos relacionados con el género. Teniendo en cuenta que los adultos universitarios emergentes se encuentran en la transición de la adolescencia a la edad adulta, una etapa en la que el cerebro aún se está desarrollando, el consumo excesivo puede comprometer los procesos neurológicos y cognitivos^(21,22)^. Esta variable tampoco fue considerada por las participantes, lo que refuerza la falta de conocimiento sobre las influencias de las características biológicas y su comportamiento de consumo.

En relación con los riesgos para la salud y el bienestar biopsicosocial, los participantes señalaron algunas consecuencias a corto plazo, como fatiga, arrepentimientos y alteraciones en las interacciones sociales. Sin embargo, los efectos a largo plazo, aunque se mencionaron, se limitaron a enfermedades hepáticas y renales, accidentes automovilísticos y conflictos familiares. El análisis sugiere una comprensión parcial de los impactos del consumo excesivo de alcohol, con escasez de información y ausencia de conexiones entre las causas, los mecanismos y los efectos del consumo. Este conocimiento fragmentado no contempla de manera clara los daños sociales, económicos y de salud pública que implica el consumo abusivo de alcohol, ni los obstáculos que representa para alcanzar los Objetivos de Desarrollo Sostenible.

El análisis de las disposiciones revela que la motivación, la curiosidad y las elecciones en relación con el consumo están directamente relacionadas con los contextos sociales, incluido el familiar. La atención centrada únicamente en la frecuencia del consumo ignora el riesgo que representa el consumo elevado en un corto período de tiempo, con poca o ninguna percepción de los efectos inmediatos y acumulativos. Sin embargo, al ser estudiantes universitarios, los participantes parecen más propensos a prácticas de consumo excesivo^(8)^, lo que refuerza la necesidad de intervenciones educativas específicas. Ante las adversidades cotidianas relacionadas con las cuestiones académicas, son comunes sentimientos como la tristeza, la ansiedad y la depresión y, en ausencia de recursos internos y externos para mantener la resiliencia, el alcohol puede utilizarse como estrategia de relajación, en un intento de disminuir el malestar emocional^(7)^. Esta realidad fue identificada en los relatos de los participantes, pero sin la profundidad necesaria para reconocer los mecanismos de vulnerabilidad y sus implicaciones a largo plazo.

Por último, considerando que la mayoría de los adultos emergentes universitarios de esta investigación trabajarán en el área de la salud y la educación, es esencial que adquieran una comprensión científica y profunda de los efectos nocivos del consumo abusivo de alcohol. Esta comprensión debe ir más allá del conocimiento empírico y, por lo tanto, debe incluir los aspectos biopsicosociales, los factores de riesgo individuales y contextuales, y las implicaciones para la salud colectiva, a fin de que puedan desempeñar un papel activo en la promoción de su salud y de las comunidades con las que interactúan.

Algunos investigadores han analizado el conocimiento de los riesgos y las unidades estándar de alcohol en cantidades específicas de cerveza, vino y bebidas destiladas. Las actitudes y prácticas de los adultos universitarios emergentes que consumen estas bebidas se producen sin conocer el patrón de consumo considerado excesivo. Los resultados obtenidos demostraron que el consumo de alcohol es una práctica habitual entre los adultos universitarios emergentes de los cursos de grado, pero que estos, incluidos los del ámbito de la salud, mostraron tener pocos conocimientos sobre sus efectos nocivos para la vida. Un estudio realizado en Sudáfrica con estudiantes universitarios, cuyo objetivo era evaluar los conocimientos, las actitudes y las prácticas relacionadas con el consumo de bebidas alcohólicas, reveló que aproximadamente un tercio de los participantes no respondió al cuestionario sobre las unidades estándar de consumo de alcohol en diferentes tipos de bebidas, como cerveza, vino y destilados. Entre los que respondieron, el 71,3% subestimó el valor correspondiente a la unidad estándar^(23)^. De manera similar, un estudio transversal realizado en Polonia con 480 universitarios de entre 18 y 35 años identificó un nivel reducido de conocimiento sobre los efectos del alcohol en la salud^(24)^.

Un estudio realizado en Portugal con adultos universitarios emergentes puso de manifiesto que, en general, tenían una puntuación baja en cuanto al conocimiento de las consecuencias negativas del alcohol para la salud y el bienestar. Sin embargo, aquellos con una puntuación más alta en conocimientos consumían más alcohol que aquellos con una puntuación más baja^(25)^. Esto puede deberse a la falta de conocimiento sobre los límites de consumo que pueden afectar a la salud. En Italia, en un estudio con 1928 estudiantes universitarios de entre 18 y 24 años, los resultados mostraron que los participantes en el estudio no eran plenamente conscientes de las consecuencias negativas del consumo excesivo de bebidas alcohólicas^(26)^. Y un estudio realizado en China presentó resultados similares^(27)^.

La mayoría de las investigaciones relacionadas con el consumo de alcohol son cuantitativas y se centran en los consumidores en general. Los aspectos diferenciadores de la mayoría de los estudios realizados sobre este tema en la presente investigación están relacionados con el enfoque cualitativo, que permitió a los adultos emergentes universitarios revisar un poco su historia de vida y reflexionar sobre la construcción de conceptos y paradigmas relacionados con el consumo de alcohol, así como repensar la repercusión de su uso en su trayectoria académica y profesional. Otro diferencial fue la población específica investigada, es decir, los adultos emergentes universitarios. Los resultados evidenciaron la relevancia de este tipo de investigación, ya que permitió profundizar el conocimiento y la concienciación sobre la percepción de los riesgos y el patrón de consumo excesivo, el impacto en su salud biopsicosocial, su bienestar y, así, replantearse su consumo, ya que surgieron preguntas sobre su comportamiento y en qué medida estaba perjudicando su rendimiento académico, así como que, si continuaban con el patrón actual, sin duda se verían afectadas sus oportunidades profesionales futuras.

## CONCLUSIÓN

Los resultados mostraron que los participantes no conocen en profundidad las repercusiones negativas y los riesgos del consumo excesivo de bebidas alcohólicas para la salud biopsicosocial y el bienestar de las personas; la cantidad de copas considerada estándar para definir el consumo excesivo, el hecho de que el cerebro aún se encuentra en desarrollo y la relación entre género y alcohol.

Como contribución al área de la salud, los resultados pueden orientar la planificación de acciones que incluyan la sensibilización y la educación en salud sobre los daños del consumo excesivo de alcohol para la salud y la calidad de vida, abordando cuestiones como: la definición de consumo excesivo; lo que constituye una dosis estándar y la cantidad de alcohol (en mililitros) correspondiente a una dosis, dependiendo del tipo de bebida; las diferencias de género; las consecuencias socioeconómicas y para la salud; los signos de dependencia del alcohol; los motivos fisiológicos por los que el alcohol está prohibido para los menores de edad, entre otros aspectos relevantes, específicamente para los adultos emergentes universitarios.

Además de la necesidad de llevar a cabo campañas de sensibilización y educación en salud, que son fundamentales para que los adultos emergentes universitarios aprendan a gestionar su tiempo y el estrés de forma saludable con el fin de mantener su salud mental. Se entiende que es importante que los profesionales de la salud, al llevar a cabo estas campañas, hagan hincapié en las razones y los medios para controlar el consumo de alcohol. Mostrar ejemplos reales de las repercusiones negativas del consumo de bebidas alcohólicas para la salud biopsicosocial y la calidad de vida a medio y largo plazo probablemente fomentará la comprensión de las advertencias y restricciones de su consumo, demostrando que estas restricciones no tienen por objeto reducir la libertad o el placer, sino evitar las consecuencias negativas para la vida y las personas que les rodean.

Entre las limitaciones del presente estudio se incluye el hecho de que la mayoría de los estudiantes universitarios son mujeres, que suelen tener una visión diferente a la de los hombres en relación con el consumo. Además, los datos se recopilaron en un contexto sociocultural específico, lo que puede influir en las percepciones de los participantes sobre el consumo excesivo de alcohol, y se llevó a cabo en una sola institución de educación superior, con un número reducido de participantes, al tratarse de una investigación cualitativa y, por lo tanto, se dio prioridad a las vivencias, experiencias y subjetividades de los estudiantes.

Por último, se considera que las brechas de conocimiento encontradas abren nuevas posibilidades para futuras investigaciones sobre el consumo de alcohol por parte de los adultos universitarios emergentes y pueden generar reflexiones sobre el papel de las políticas relativas al consumo de alcohol y otras drogas, así como sobre la función social de las universidades, con el fin de proporcionar una mayor calidad de vida a esta población específica que, en su mayoría, pasa a formar parte de la fuerza productiva del país.

## Data Availability

Los datos que sustentan este estudio han sido depositados en el repositorio SciELO Data, el cual puede ser consultado a través de la siguiente dirección electrónica https://data.scielo.org/dataset.xhtml?persistentId=doi:10.48331/SCIELODATA.RU7N8N.
